# Synergistic review of automation impact of big data, AI, and ML in current data transformative era

**DOI:** 10.12688/f1000research.161477.1

**Published:** 2025-03-03

**Authors:** Swastik Rath, Manjusha Pandey, Siddharth Swarup Rautaray

**Affiliations:** 1 Computer Science and Engineering, Kalinga Institute of Industrial Technology, Bhubaneswar, Odisha, 751003, India

**Keywords:** Big data analytics, artificial intelligence, machine learning, automation, predictive analytics, data-driven decision-making, risk management, fraud detection, consumer behavior analysis, electronic health records, cyber security [5], Sustainability

## Abstract

The convergence of automation, big data analytics (BDA), artificial intelligence (AI), and machine learning (ML) has ushered in a new era of technological advancement, reshaping industries, and societies worldwide. This review research work delves into the transformative impact of these technologies, focusing on their applications across various sectors. The study covers six key sectors: healthcare, banking, finance, retail, real estate, and agriculture, highlighting how these industries leverage automated systems and data analytics to enhance operations, manage risks, and improve decision-making processes. Drawing results from over 1,000 research papers and categorizing them into 100 key studies specifics, this survey-based review underscores the critical role of big data in enabling predictive analytics, improving outcomes, and driving innovation across sectors. The review research work explores how industries utilize vast data volumes from diverse sources to derive actionable insights, forecast trends, and optimize processes. Key applications included in the review are from the domains of disease prediction and electronic health record management in healthcare , fraud detection and credit risk assessment in banking and finance, consumer behavior analysis and inventory optimization in retail, market trend forecasting in real estate, and disaster risk management in agriculture. The paper also discusses the challenges including data quality, scalability, and privacy paving way towards future directions of big data analytics, emphasizing the need for machine-independent solutions, data security, and ethical considerations in the evolving landscape of data-driven decision-making.

## 1. Introduction

This review research work offers a thorough examination of how automation has led to data proliferation across various industries, focusing on the effects of big data analytics (BDA), artificial intelligence (AI), and machine learning (ML) technologies. ^1^ The research covers six crucial sectors: healthcare, banking, finance, retail, real estate, and agriculture, highlighting their reliance on automated systems to enhance operations, manage risks, and improve decision-making processes. In the healthcare field, AI and BDA are revolutionizing disease forecasting, electronic health record (EHR) administration, and supply chain ^2^ performance. The banking and finance industries employ automation for detecting fraudulent activities, evaluating credit risks, and bolstering cybersecurity measures. Retail businesses benefit from automation through improved productivity, analysis of consumer behavior, ^3^ and prediction of sales trends. The real estate market utilizes predictive analytics to identify market patterns, particularly during the COVID-19 crisis, ^4^ while the agricultural sector leverages BDA to boost operational efficiency, predict area specific crops/products and manage disaster risks.

The generation of vast data volumes from diverse sources, such as EHRs, transactional records, and climate data, necessitates sophisticated big data technologies ^5^ for effective processing and insights. Data is recognized as a valuable resource, offering historical insights and predictive capabilities across these sectors. This survey draws from over 1,000 research papers, categorizing 100 key studies to underscore the transformative role of big data in enabling predictive analytics, improving outcomes, ^6^ and driving innovation in these industries.

### 1.1 Automation

In this section, the research work utilizes different domains which have different types and of requirements of automation,

1.1.1 **Healthcare:** These studies automate healthcare management through big data ^7^ and artificial intelligence, improving disease prediction, supply chain efficiency, and patient care. ^8^ Tools such as machine learning, natural language processing, and deep learning streamline electronic health record analysis, ^9^ risk assessment, decision making, ^10^ transforming healthcare practices, and outcomes. ^11^ The transformative potential of big data in healthcare, focusses on improving system efficiency, patient outcomes, and personalized care. ^12^ They identify challenges in data quality, integration, and ethics, recommending improved governance, interdisciplinary collaboration, and standardized frameworks to enhance big data’s effectiveness in healthcare applications. ^13^ The integration of big data in digital healthcare provides lessons and recommendations for leveraging big data to improve patient outcomes, streamline operations, and advance personalized medicine in primary healthcare settings. Applications of big data analytics in health care emphasizes big data’s potential to revolutionize healthcare delivery, improve patient outcomes, and address challenges such as data security and ethical considerations. ^14^

1.1.2 **Banking:** Automation in banking leverages big data and artificial intelligence for fraud detection, credit risk assessment, and operational optimization. Machine learning models and data analytics platforms enhance real-time insights, cybersecurity, financial decision-making, and driving efficiency across the sector.

1.1.3 **Finance:** In finance, automation driven by big data, artificial intelligence, and machine learning optimizes risk mitigation, credit evaluation, and financial analysis. IoT integration, data-driven processes, and advanced algorithms streamline financial services, sustainability assessments, and innovation, thereby transforming the industry’s operational landscape. ^15^

1.1.4 **Retail:** These studies automate retail operations using big data analytics, enhancing productivity, fraud detection, and customer behavior analysis. Big data-driven tools transform traditional retail, predict sales, and analyze the impact of external factors such as COVID-19.

1.1.5 **Real estate:** Automation in real estate leverages big data and predictive analytics to forecast market trends, particularly during the COVID-19 pandemic.

1.1.6 **Agricultural:** These papers automate agricultural processes and disaster management using big data analytics, driving operational efficiency and informed decision-making.

## 2. Machine dependency in Big Data

In this section, the research work utilizes different domains which have Machine Dependency in Big data

### 2.1 Machine dependency in Healthcare

**Figure 1.**
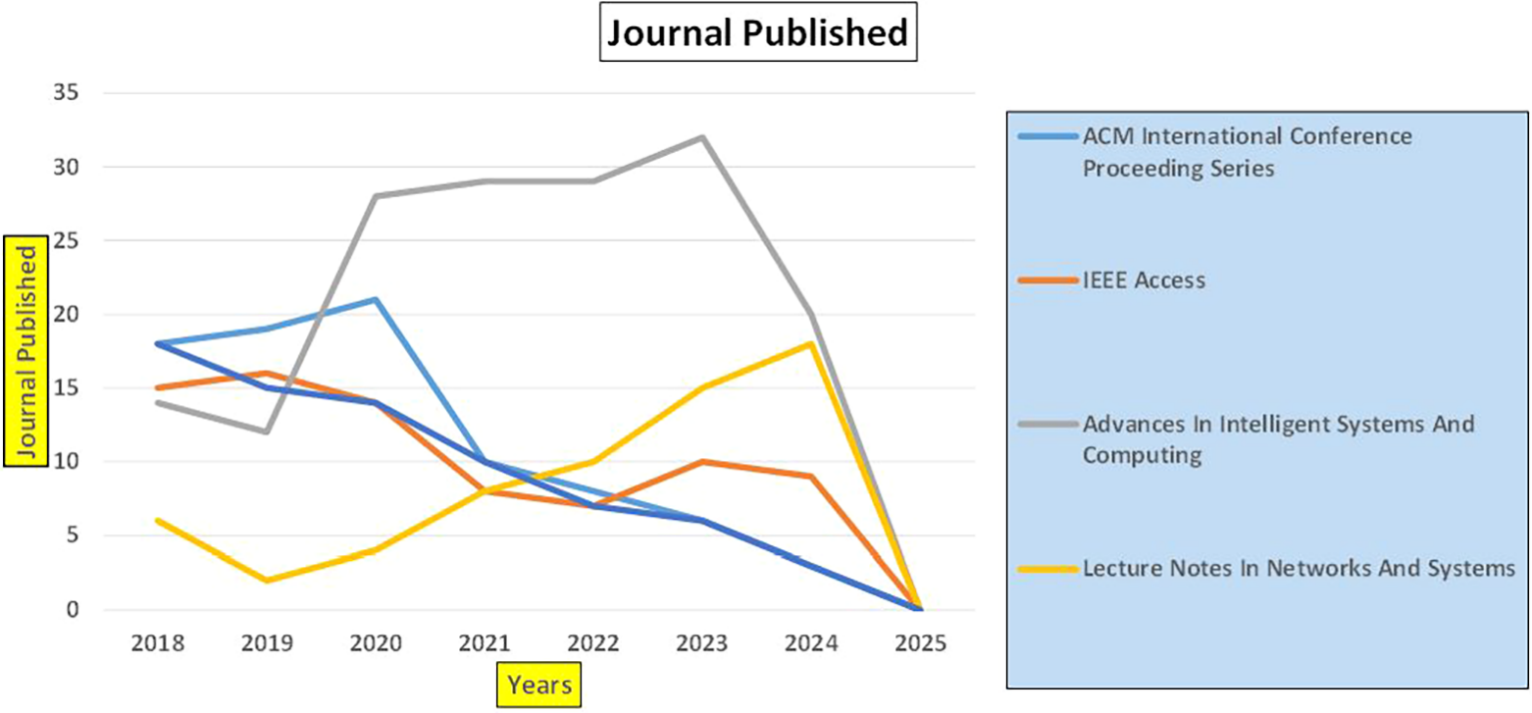
Healthcare Research Papers Published by Source (2018-2025).

These studies demonstrate a significant reliance on machine learning, artificial intelligence, and big data analytics for predictive ^16^ modeling, risk assessment, and decision-making in healthcare. They employ deep learning, natural language processing, and advanced data processing systems to automate complex tasks such as electronic health record analysis, disease prediction, and healthcare supply chain management ( Figure 1). ^17^

**Figure 2.**
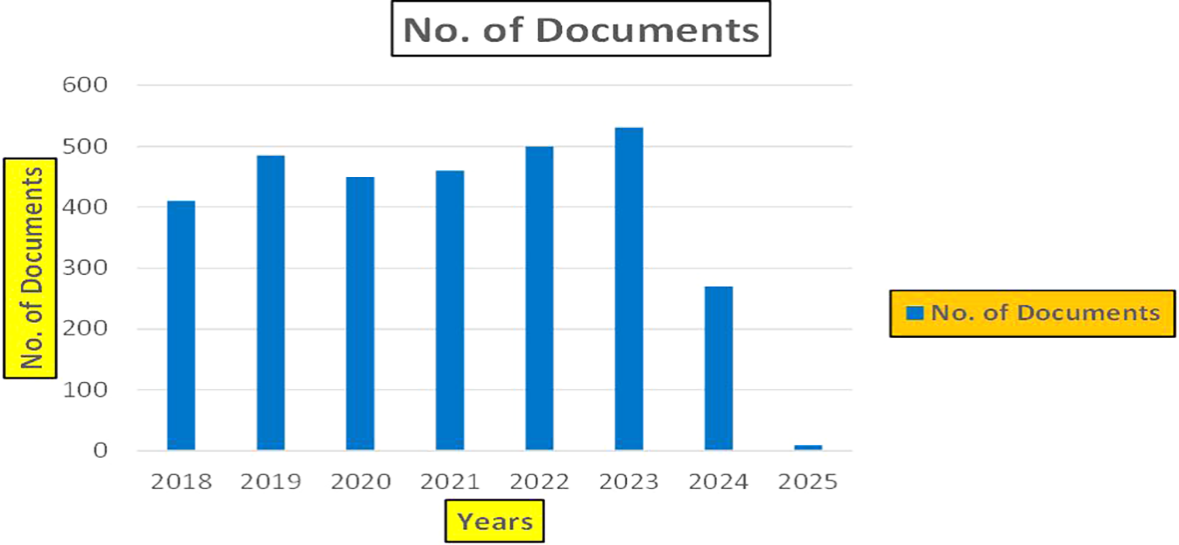
Healthcare Research Publications (2018-2025): Number of Papers Published.

Machine independence in healthcare refers to the capacity of healthcare systems ^18^ to operate effectively without dependence on specific hardware or software platforms. This enhances system flexibility, scalability, and longevity by mitigating vendor lock-in ( Figure 2).

In healthcare, machine-independent solutions frequently utilize open-source technologies, cloud-based infrastructures, and standardized data formats. Through this approach, hospitals and care providers can integrate AI and big data analytics into their workflows while maintaining interoperability with various systems, such as EHR platforms from multiple vendors. This promotes collaboration, data sharing, and system upgrades without incurring costly disruptions.

**Figure 3.**
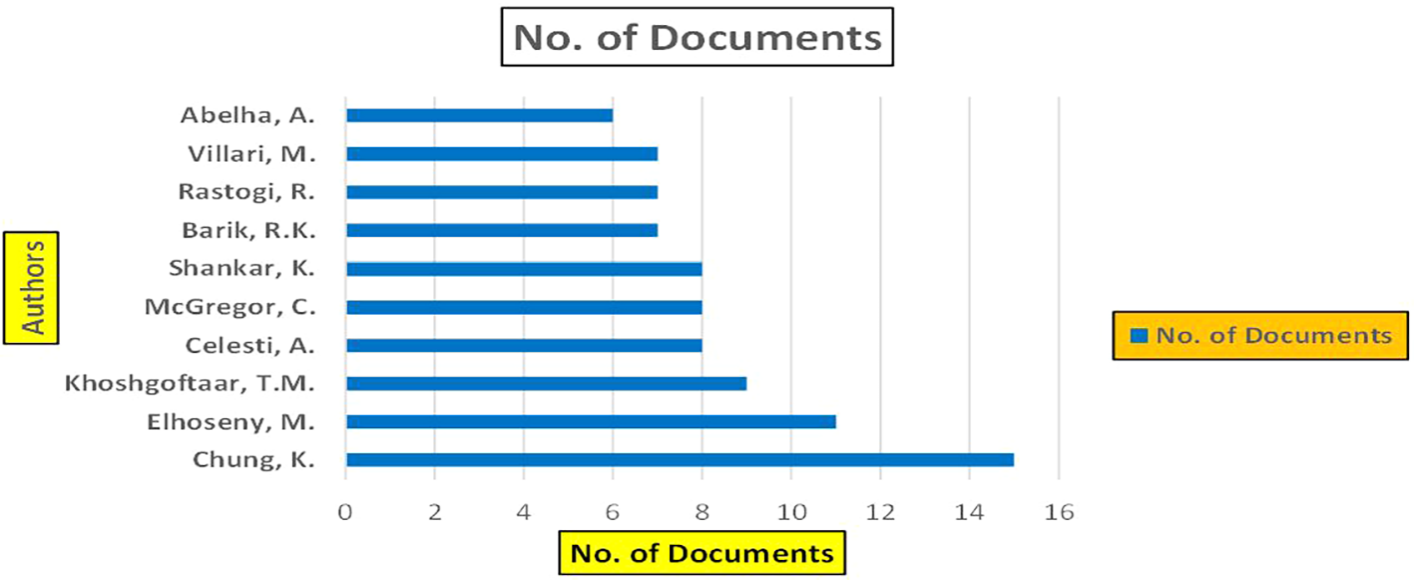
Healthcare Research Papers by Affiliation (2018-2025): Publication Trends.

Machine independence is particularly crucial as healthcare progresses towards Industry 4.0, ^19^ where data-driven approaches are central. ^20^ Ensuring that AI and big data tools ^21^ are machine-independent facilitates seamless integration across diverse healthcare environments, supporting a more robust, adaptable, and accessible healthcare system. It also facilitates the adoption of innovative technologies and enhances patient outcomes across various clinical settings ( Figure 3).

**Figure 4.**
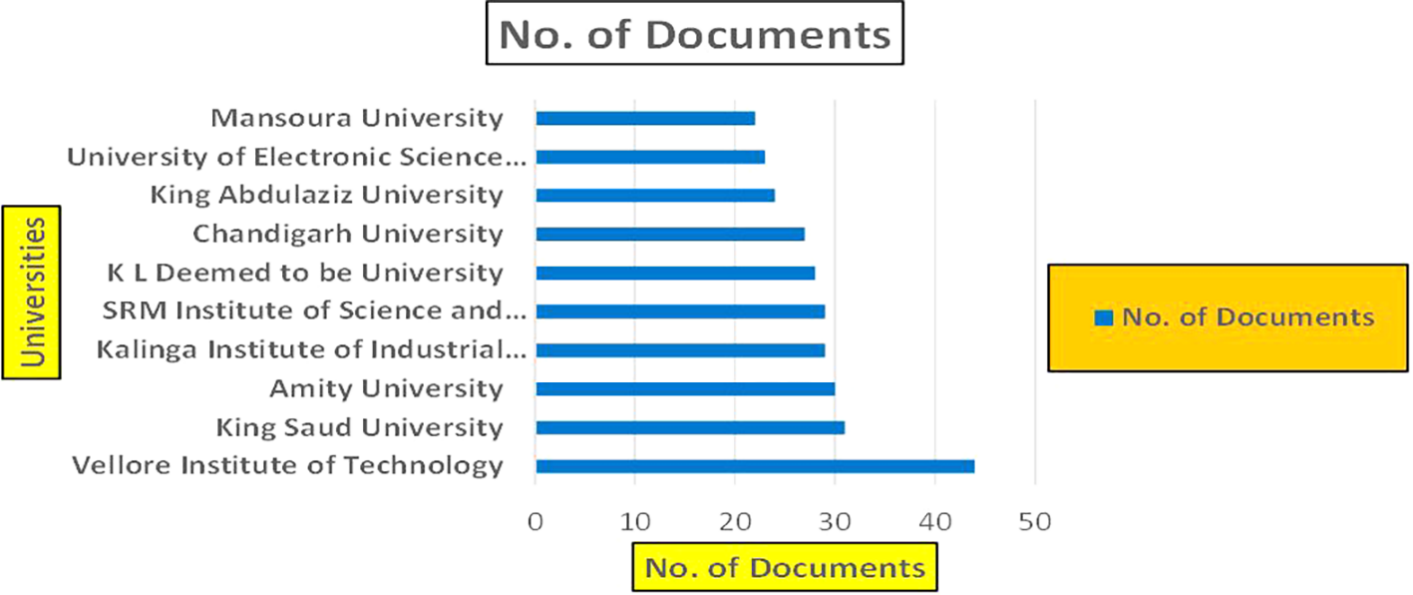
Healthcare Research Papers by Universities (2018-2025): Publication Trends.

Machine independence in healthcare is essential for ensuring that critical systems and technologies can adapt to rapidly changing environments without being constrained by specific hardware or software. This characteristic is particularly significant in the context of big data analytics and artificial intelligence (AI), where data sources and computational requirements vary across healthcare providers. Machine-independent systems can operate on diverse infrastructure configurations, whether on-premise, in the cloud, or across hybrid systems, promoting flexibility and scalability ( Figure 4).

In healthcare, where integration and interoperability are crucial, machine independence enables different institutions to share, access, and process large volumes of data across disparate systems. This flexibility supports enhanced decision-making, expedited diagnoses, and more personalized treatment plans by facilitating the utilization of advanced AI algorithms and big data analytics, irrespective of the platform.

It emphasizes the transformative potential of big data in areas such as diagnostics, disease prediction, personalized medicine, and operational management. The authors discuss how big data supports evidence-based decision-making, improves patient outcomes, and streamlines healthcare processes. ^22^

**Figure 5.**
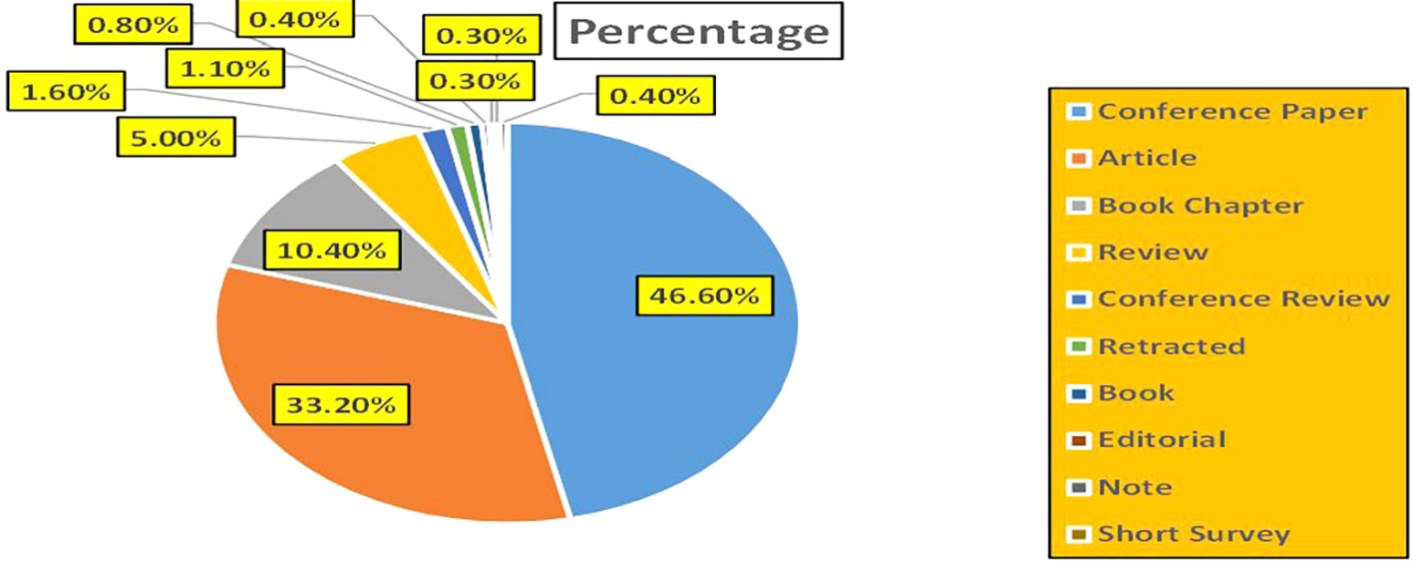
Healthcare Research Papers by Source (2018-2025): Publication Trends.

A significant advantage of machine independence is its capacity to foster innovation without being limited by existing systems. Healthcare facilities can incorporate cutting-edge technologies such as natural language processing (NLP), computer vision, or machine learning algorithms into their operations, regardless of the underlying IT infrastructure. This allows healthcare organizations to evolve alongside technological advancements without costly overhauls or disruptions to patient care ( Figure 5).

Machine independence also contributes to more robust and resilient healthcare infrastructures, enabling organizations to diversify their technology stack, enhance system security, and ensure continuity of operations even in the event of specific hardware or software failures.

### 2.2 Machine dependency in Banking

**Figure 6.**
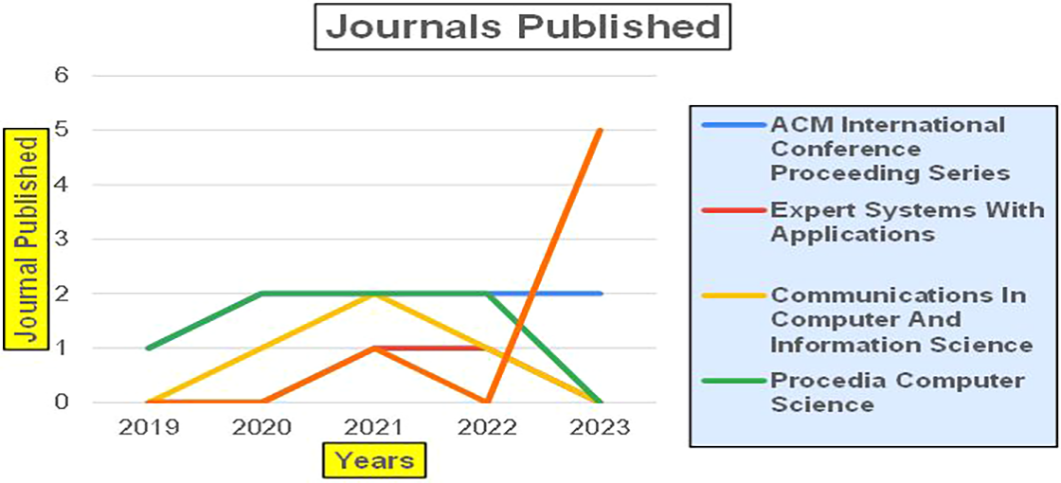
Banking Research Papers Published by Source (2019-2024).

Machine learning models and big data analytics ^23^ play a central role in automating fraud detection, credit risk assessment, and real-time banking insights. These studies emphasize the significance of artificial intelligence and data-driven platforms in enhancing banking operations, cybersecurity, and financial decision making ( Figure 6).

**Figure 7.**
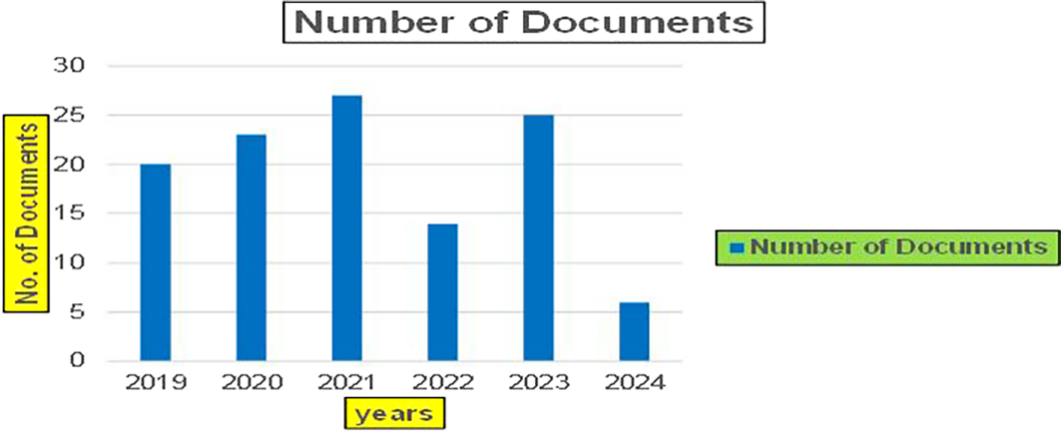
Banking Research Publications (2019-2024): Number of Papers Published.

Machine dependency in big data analytics, especially in the context of banking and finance, plays a pivotal role in achieving the scale, speed, and complexity required for modern applications such as fraud detection, credit risk assessment, and cybersecurity. The machine dependency aspect refers to how heavily these analytics systems rely on computational power, advanced algorithms, and data storage infrastructure to deliver accurate and timely results ( Figure 7)

**Figure 8.**
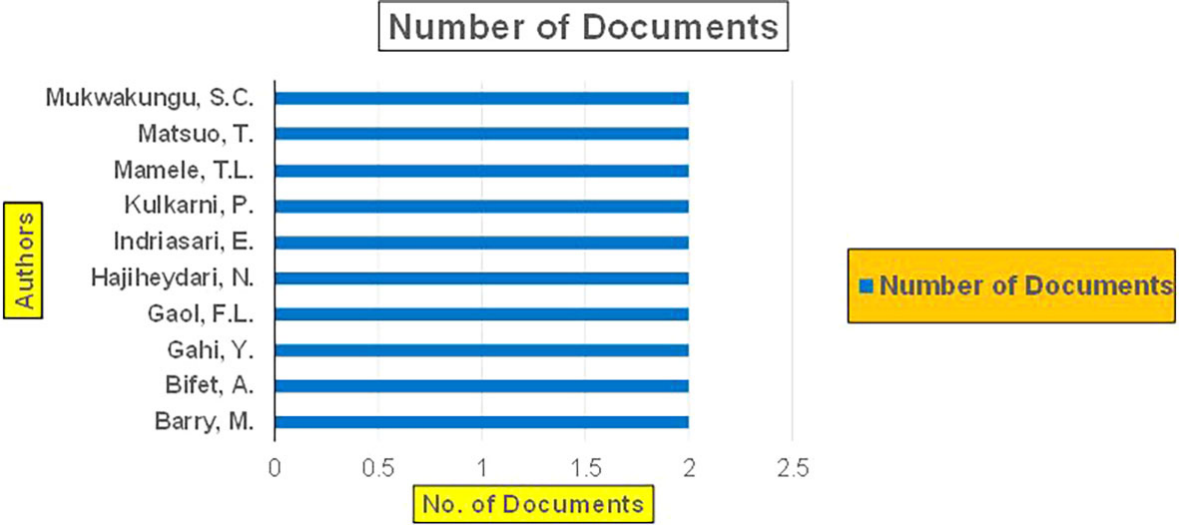
Banking Research Papers by Affiliation (2019-2024): Publication Trends.

In **credit/debit card fraud detection**, ^24^ especially in real-time, machine learning models such as decision trees, neural networks, and support vector machines (SVM) require powerful computing resources to process massive amounts of transactional data at high speeds. The dependency on machines ensures that fraud can be detected before it causes substantial financial damage ( Figure 8).

**Figure 9.**
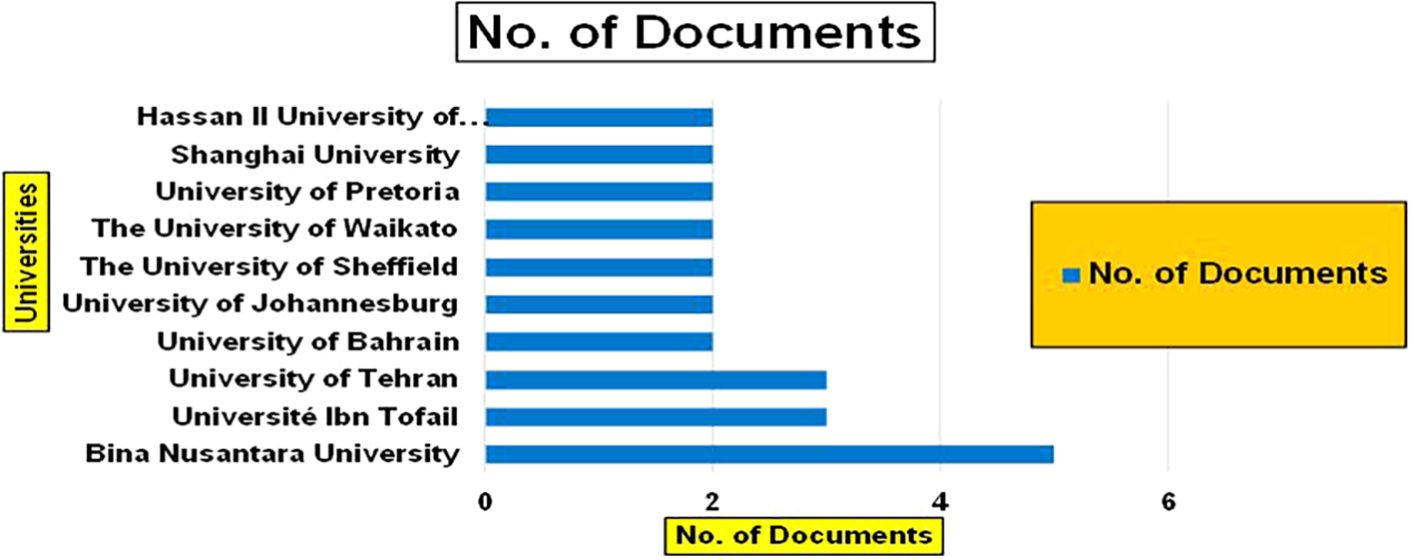
Banking Research Papers by Universities (2019-2024): Publication Trends.

For **credit/debit card-not-present fraud detection**, ^25^ machine dependency becomes even more crucial. The large-scale analysis of transactional metadata (such as geolocation and device fingerprints) relies on big data frameworks like Hadoop and Spark, alongside cloud-based computing resources. These systems enable parallel processing of vast datasets, ensuring that analytics models can detect suspicious behavior in real-time ( Figure 9).

In **credit risk assessment** and **quantifying cybersecurity risks**, ^26^ machine learning algorithms such as decision trees and SVMs are computationally intensive, especially when analyzing complex and large datasets. Machine dependency in these tasks ensures that the models can run continuously and make decisions based on real-time data streams, which is critical for timely risk assessments.

**Figure 10.**
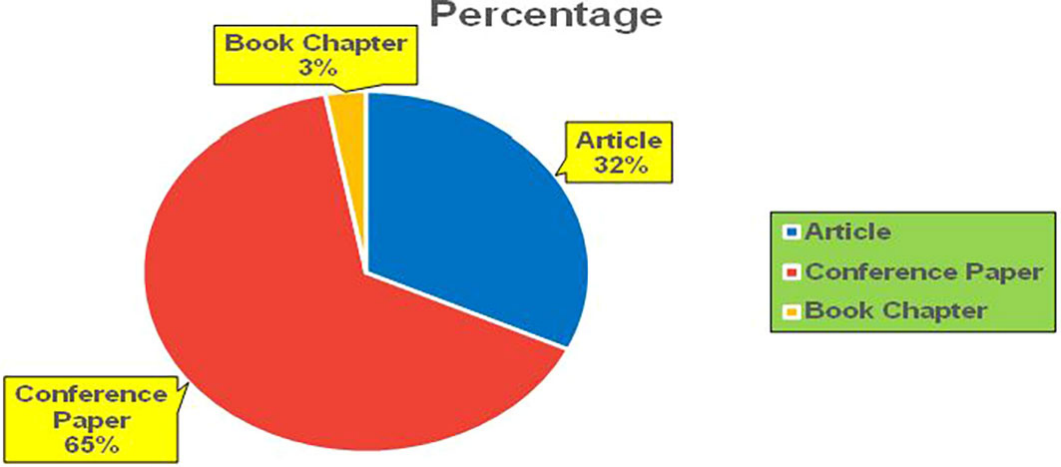
Banking Research Papers by Source (2019-2024): Publication Trends.

The overall **dynamics of big data analytics in the banking sector** ^27^ show an increasing reliance on AI and machine learning models that require not just data but also computing power to function effectively. With the imperatives of big data in finance, ^28^ the integration of **cloud infrastructure**, **distributed computing systems**, and **advanced hardware** (such as GPUs) becomes essential to run algorithms at the scale needed for financial applications ( Figure 10).

In summary, machine dependency is foundational to enabling big data analytics systems to handle large, complex datasets, ensuring real-time insights and decision-making in the banking and finance sector. ^29^

### 2.3 Machine dependency in Finance

**Figure 11.**
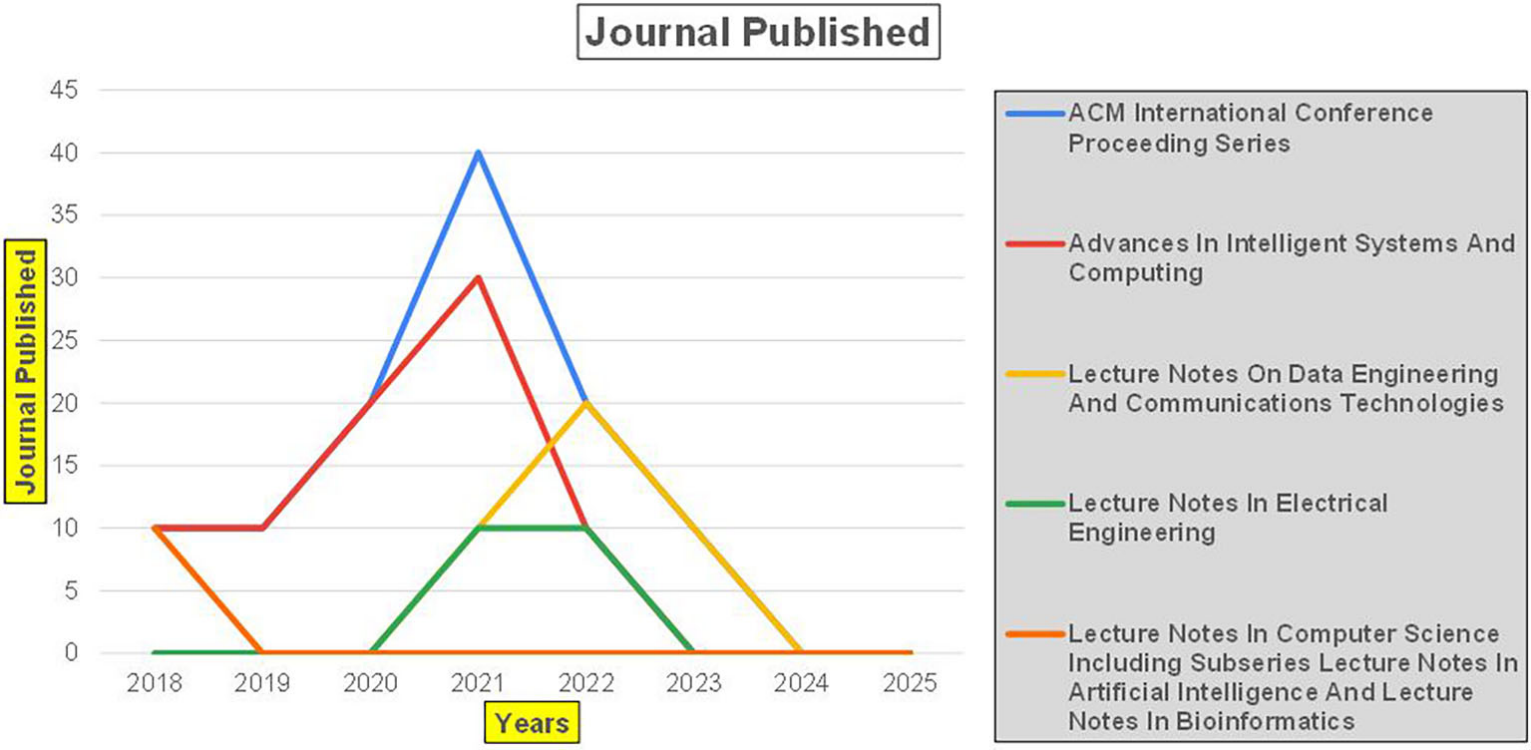
Finance Research Papers Published by Source (2018-2025).

Big data, artificial intelligence, and machine learning facilitate the automation of financial processes such as risk mitigation, fraud detection, and credit evaluation. These studies highlight the integration of the IoT, data-driven analysis, and machine learning algorithms to transform financial operations, ^30^ sustainability analysis, and digital finance ^31^ services ( Figure 11).

**Figure 12.**
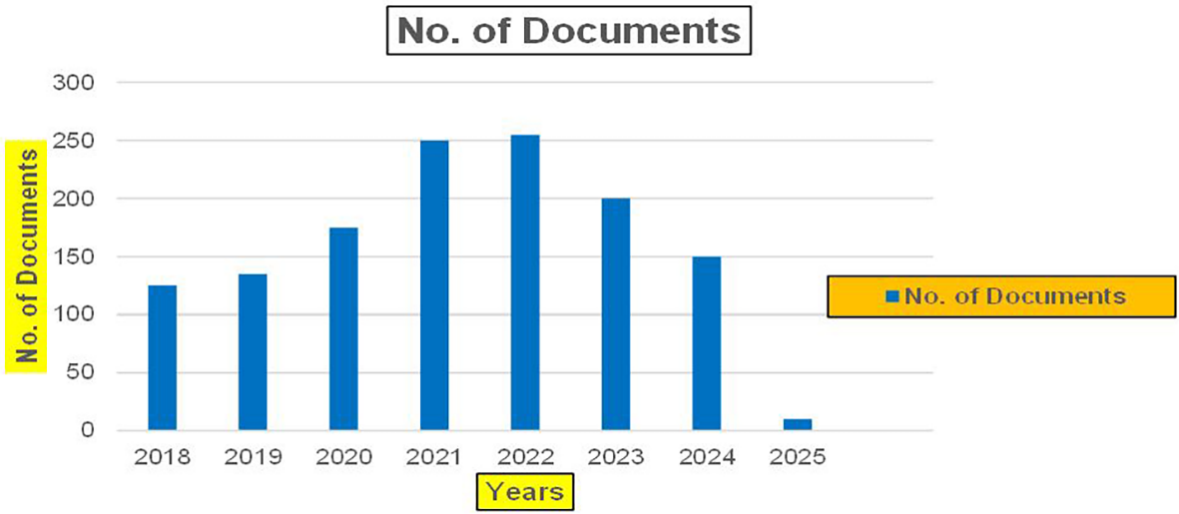
Finance Research Publications (2018-2025): Number of Papers Published.

Machine dependency in big data within the finance sector refers to the reliance on specific hardware, software, and computing infrastructure to effectively process, analyze, and utilize large datasets for financial decision-making. Big data analytics in finance often necessitates powerful computational resources, specialized software, and cloud platforms that support extensive data storage and high-speed processing. Machine learning algorithms employed for tasks such as risk mitigation, fraud detection, and credit evaluation depend on specific systems and frameworks for training models and making predictions ( Figure 12).

**Figure 13.**
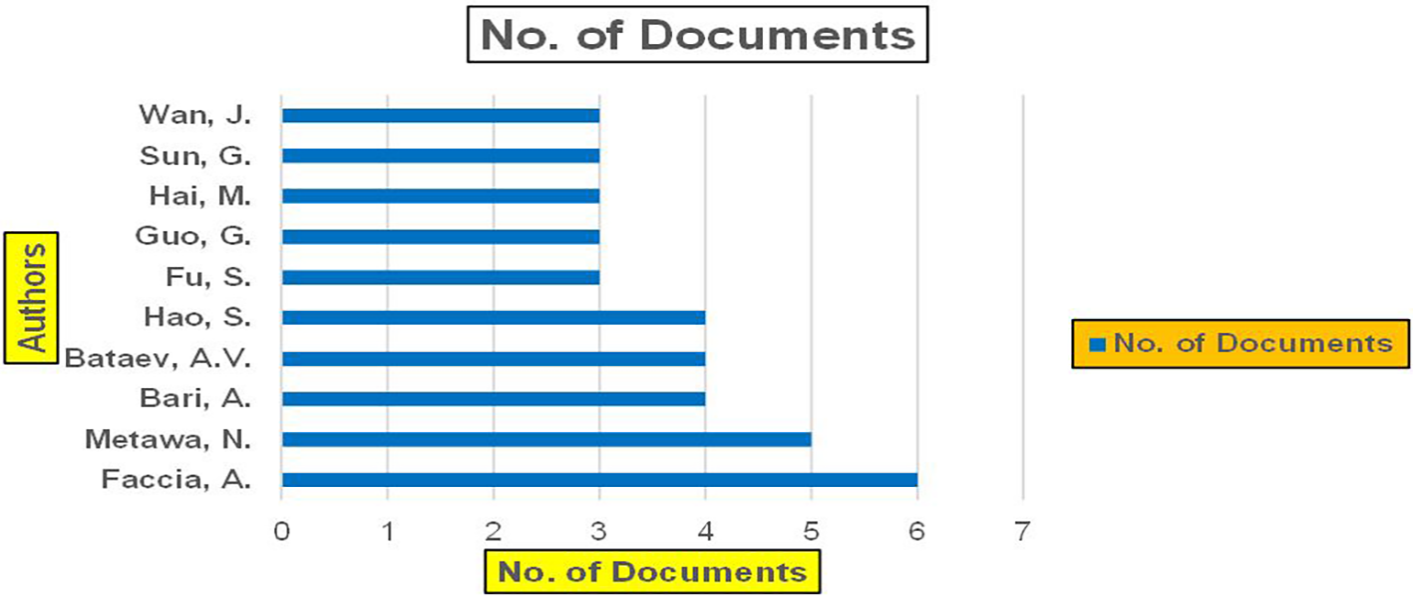
Finance Research Papers by Affiliation (2018-2025): Publication Trends.

The financial industry frequently encounters challenges due to machine dependency, such as reliance on proprietary software for algorithmic trading, data analysis, or fraud detection, which constrains flexibility and interoperability across different systems. Financial institutions may become constrained within specific vendor ecosystems, diminishing their ability to switch providers or integrate new technologies without substantial costs ( Figure 13).

**Figure 14.**
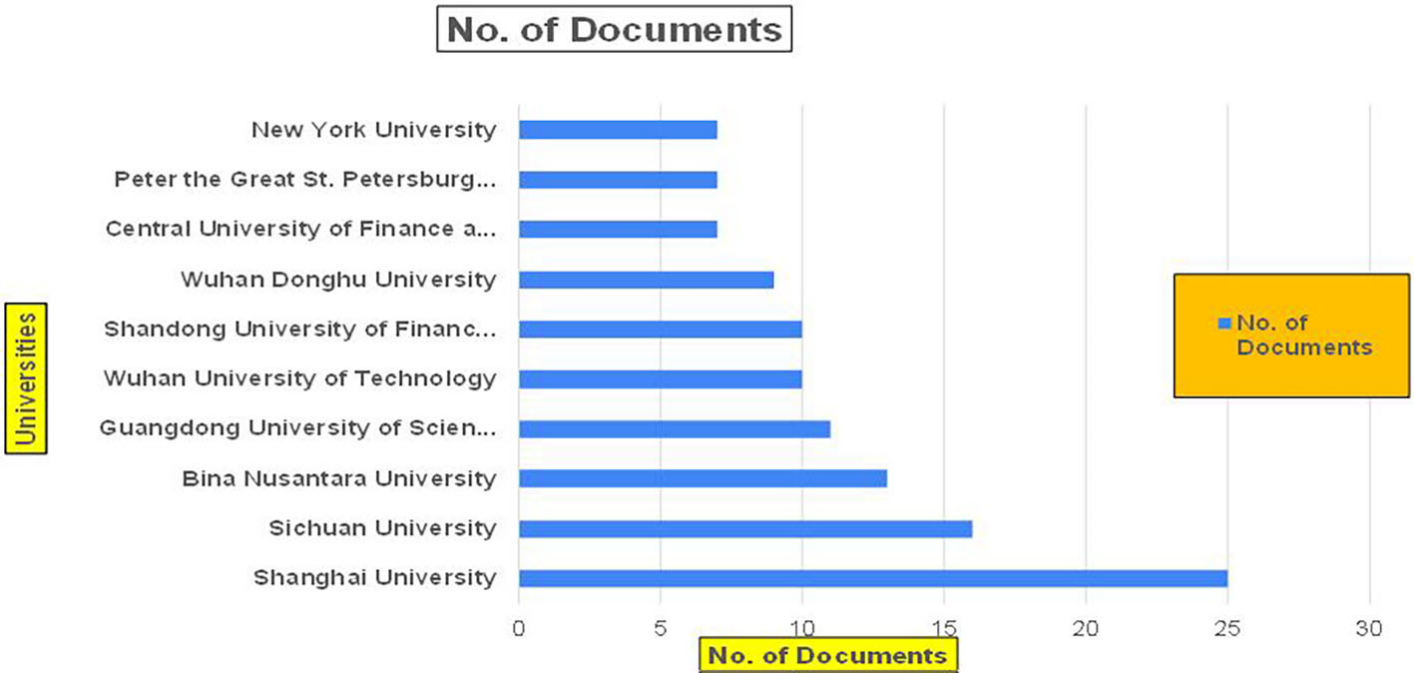
Finance Research Papers by Universities (2018-2025): Publication Trends.

Machine dependency also raises concerns regarding data security, as financial data must often be stored and processed on particular machines or cloud environments, potentially increasing the risk of cyber threats if systems are compromised. While machine learning and artificial intelligence enhance financial processes, the dependency on particular machines and infrastructure can impede innovation and adaptability unless measures are taken to adopt more platform-agnostic solutions, such as open-source software or cloud-agnostic platforms ( Figure 14).

Machine dependency in big data in finance ^32^ refers to the reliance on specific computational infrastructure, algorithms, and software systems to process and analyze massive amounts of financial data. As financial institutions increasingly adopt big data analytics and machine learning for risk mitigation, fraud detection, and credit evaluation, machine dependency can become a limiting factor. This reliance on proprietary hardware, software, or algorithms often results in inflexibility, impeding adaptation to new technologies or integration with other systems. ^33^

**Figure 15.**
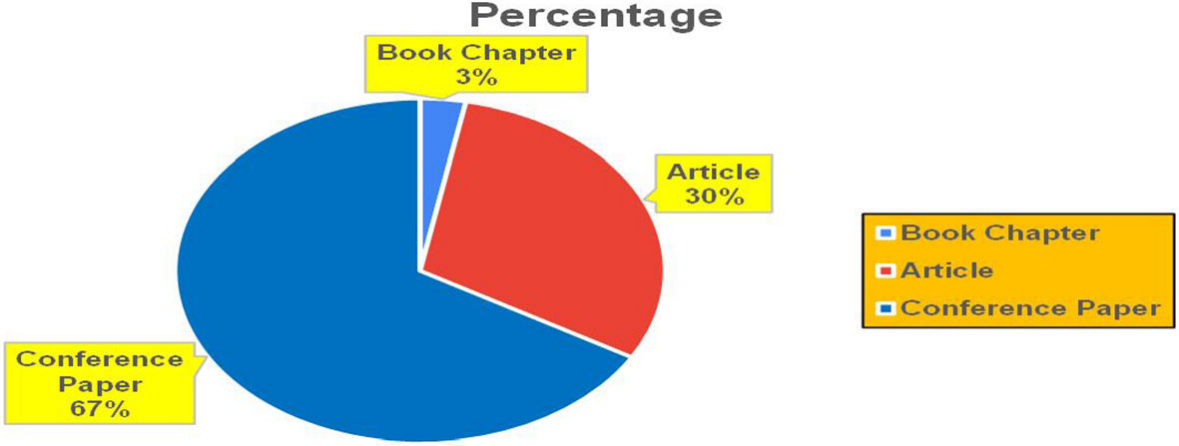
Finance Research Papers by Source (2018-2025): Publication Trends.

For instance, risk mitigation and fraud detection models may rely heavily on certain machine learning frameworks that are optimized for specific hardware environments. In such cases, transitioning to alternative platforms or integrating with different systems might necessitate costly re-engineering. Similarly, credit evaluation systems driven by big data and the Internet of Things (IoT) may depend on custom-built architectures, thereby restricting scalability or interoperability across different platforms ( Figure 15).

### 2.4 Machine dependency in Retail

**Figure 16.**
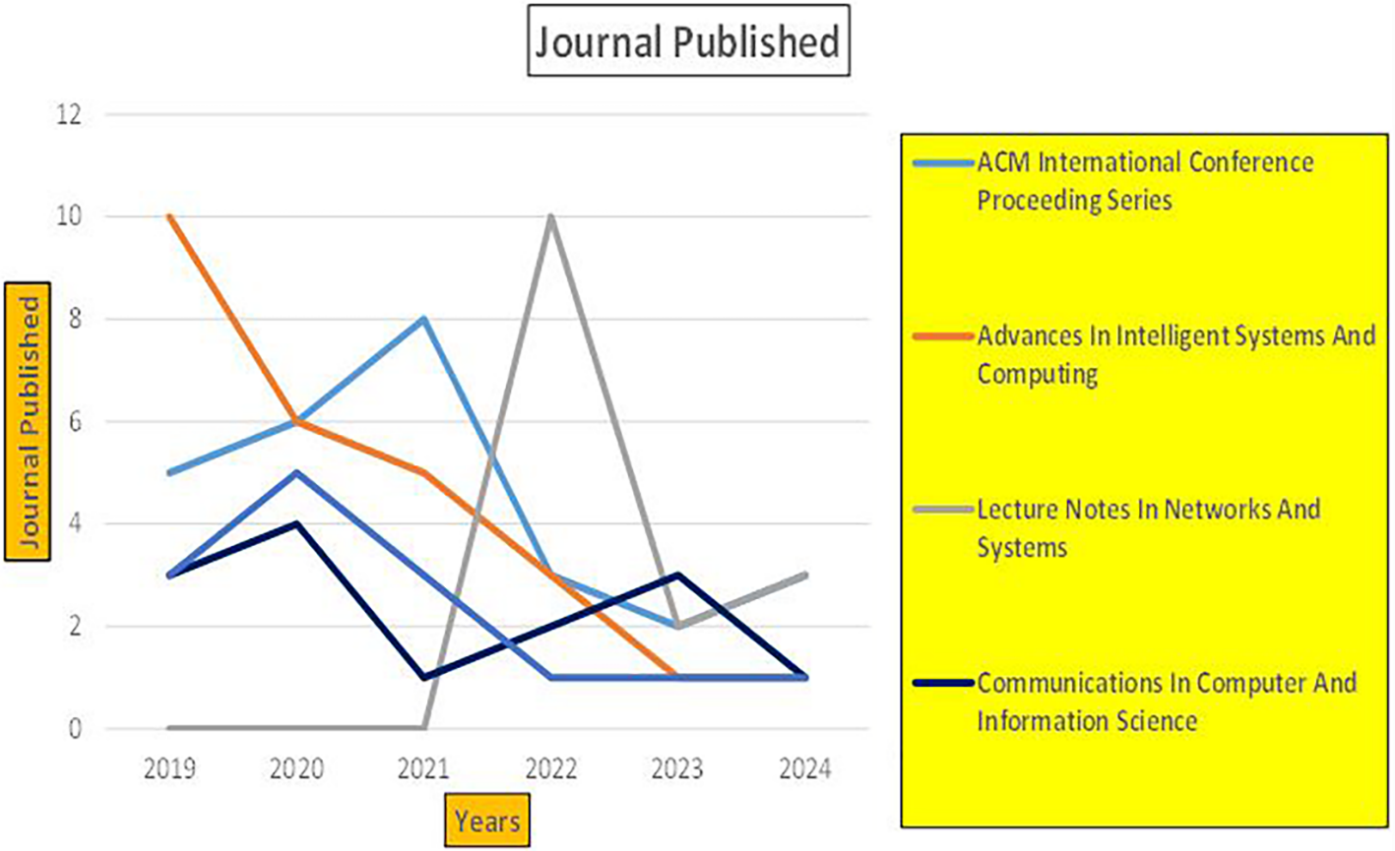
Retail Research Papers Published by Source (2019-2024).

Retail-related studies utilize big data technologies, including advanced analytics, machine learning models, and data fusion techniques, to optimize retail store productivity, detect fraud, predict sales trends, and analyze consumer behavior ( Figure 16).

**Figure 17.**
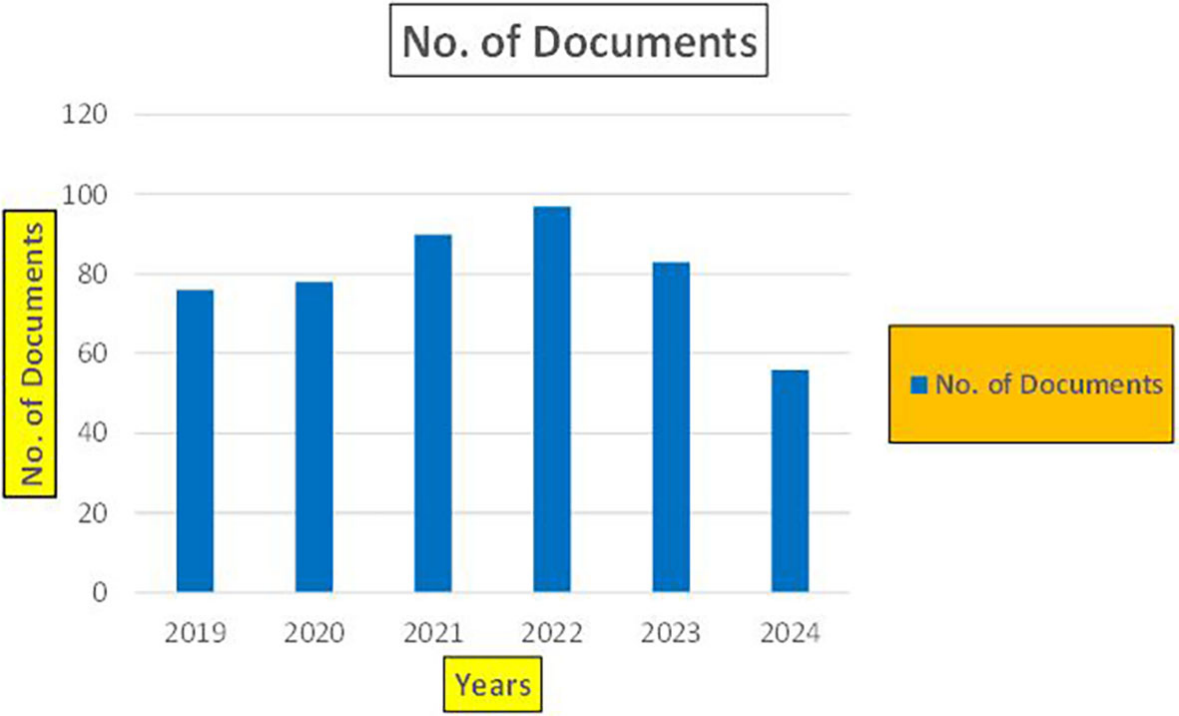
Retail Research Publications (2019-2024): Number of Papers Published.

Machine dependency in the context of big data technologies in retail refers to the reliance on specific hardware, software, or algorithms that can create barriers to flexibility, scalability, and innovation. As retail companies increasingly adopt big data analytics for various applications, such as enhancing productivity, combating fraud, and understanding consumer behavior, this dependency can significantly impact their operational efficiency and adaptability ( Figure 17).

**Figure 18.**
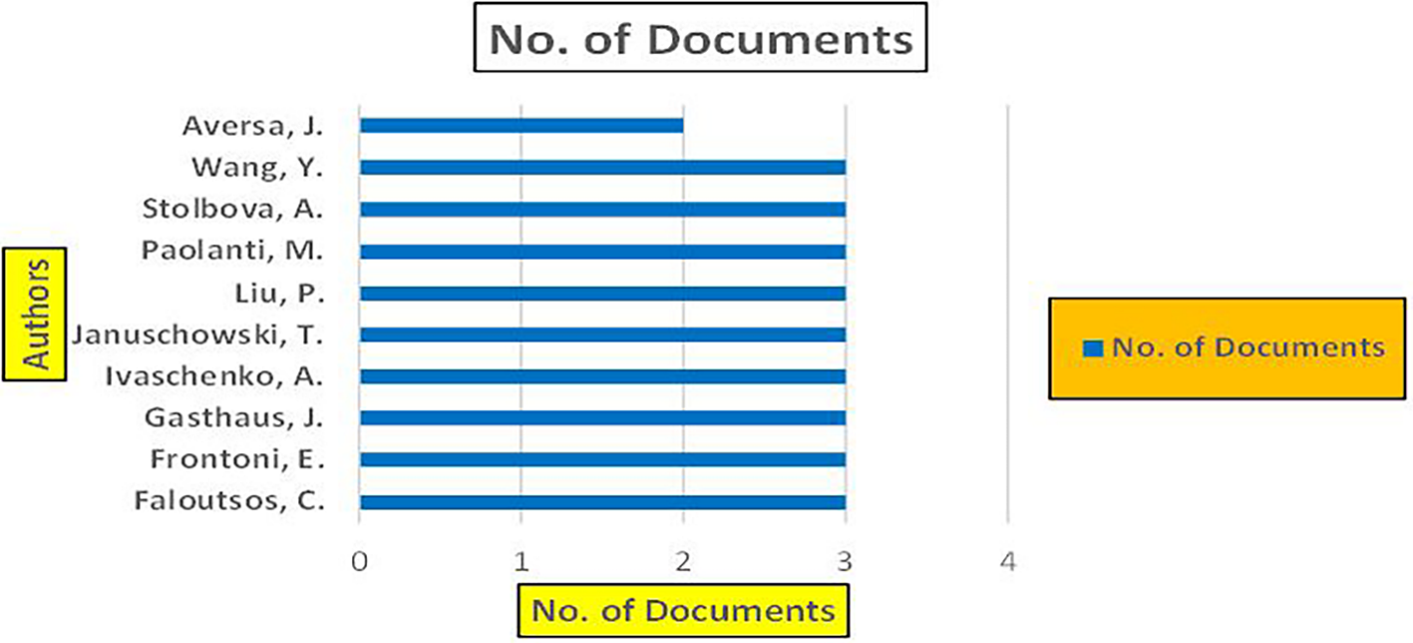
Retail Research Papers by Affiliation (2019-2024): Publication Trends.

For instance, many retail analytics ^34^ solutions require robust computational infrastructure to process large datasets effectively. This often leads to reliance on specific machine learning frameworks and databases optimized for hardware configurations. Such dependencies can hinder a retailer’s ability to integrate newer technologies or switch to alternative systems, resulting in increased costs and longer implementation times. For example, in analyzing the sales data of Bigmart ^35^ outlets, reliance on specific analytics software may limit the organization’s ability to pivot quickly in response to market changes ( Figure 18).

**Figure 19.**
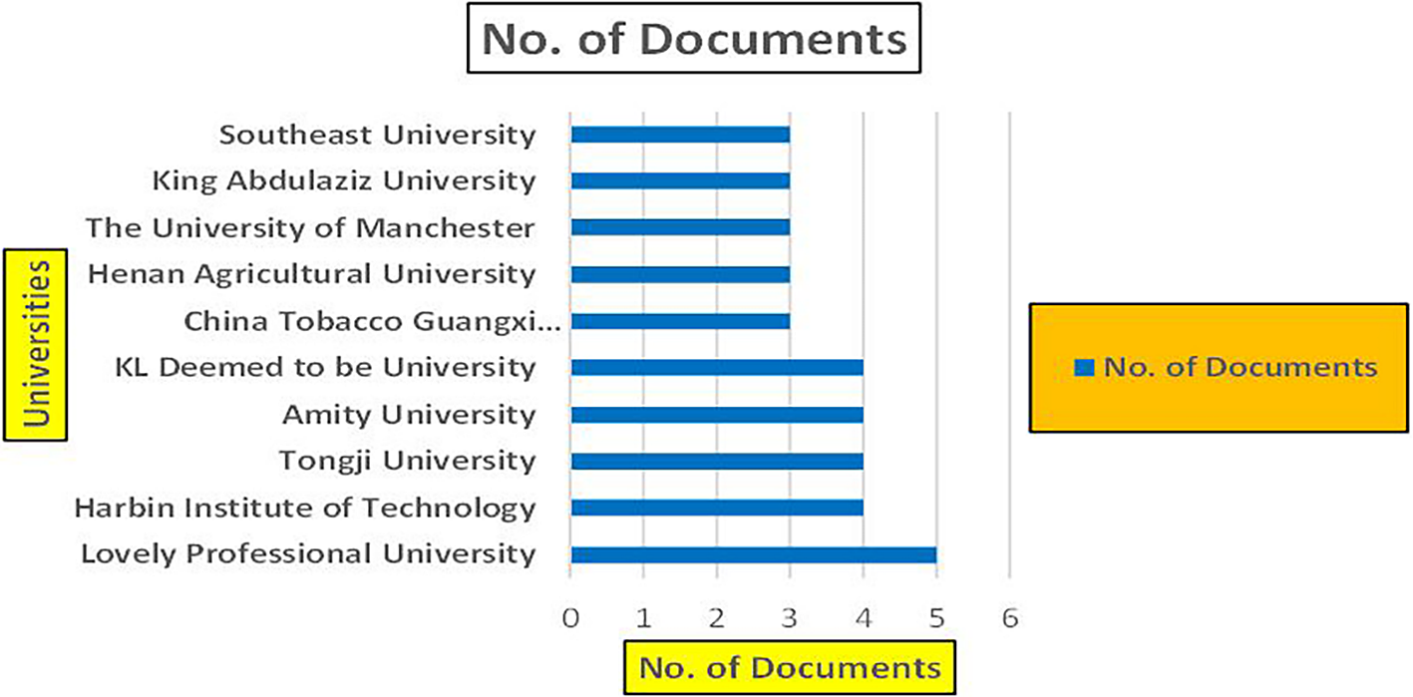
Retail Research Papers by Universities (2019-2024): Publication Trends.

Additionally, the use of big data to enhance data envelopment analysis (DEA) of retail store productivity ^36^ illustrates another aspect of machine dependency. If the DEA models are built on specific platforms, retailers may face challenges in scaling these models or adapting them to different data sources, restricting their capacity to evaluate productivity effectively across diverse retail environments ( Figure 19).

Fraud detection systems, which leverage big data analytics to combat retail fraud, ^37^ are also susceptible to machine dependency. Often, these systems are built on tailored algorithms that operate best within certain frameworks. This can limit retailers’ ability to update or adapt their fraud detection capabilities as new threats emerge, ultimately compromising security.

**Figure 20.**
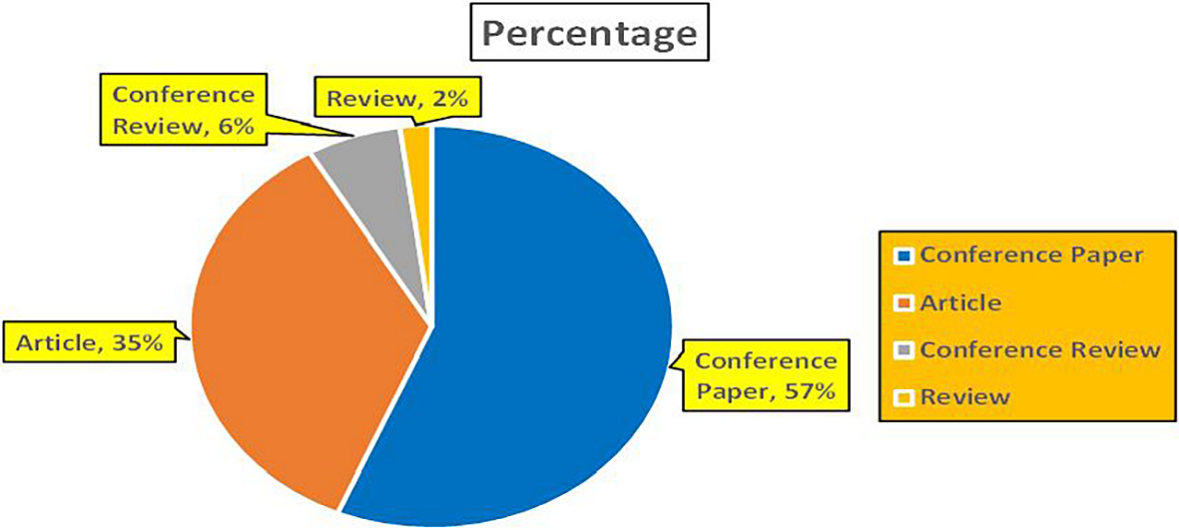
Retail Research Papers by Source (2019-2024): Publication Trends.

The analysis of consumer behavior using big data further underscores the risks of machine dependency. Retailers may become overly reliant on certain data sources, such as social media ^38^ or point-of-sale data, processed through specific analytics platforms. This can result in a narrow view of consumer insights, limiting the retailer’s ability to innovate and respond to shifting consumer preferences ( Figure 20).

Moreover, the transformation of traditional retail ^39^ in the era of big data often involves integrating multiple data streams, including demographic data, sales data, and online behavior. If retailers are locked into specific technologies, they may struggle to achieve the necessary interoperability and flexibility to leverage these diverse data sources effectively.

In conclusion, while big data technologies present significant opportunities for retail companies, the potential for machine dependency poses challenges that can inhibit innovation, limit adaptability, and increase operational costs. ^40^ Retailers need to adopt machine-independent solutions to ensure flexibility in their big data initiatives and maintain a competitive edge in a rapidly evolving market. ^41^

### 2.5 Machine dependency in Real Estate

**Figure 21.**
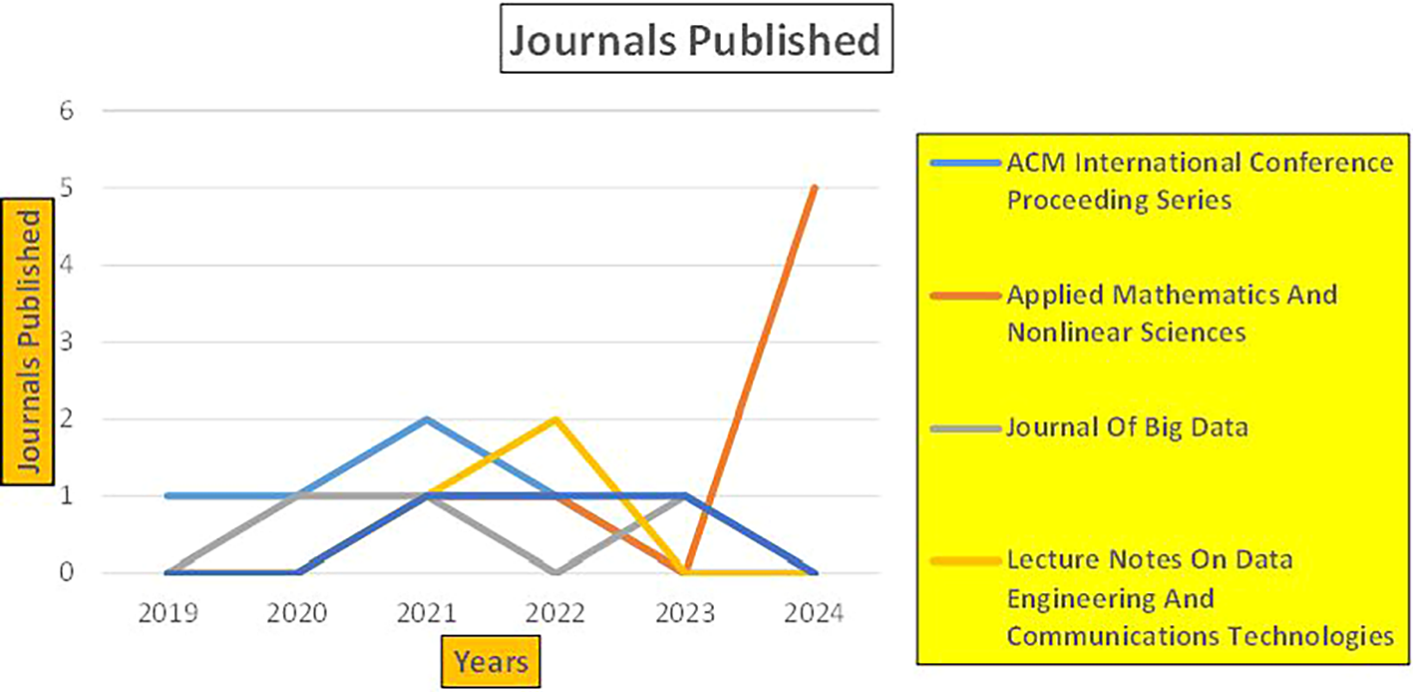
Real Estate Research Papers Published by Source (2019-2024).

The studies on forecasting commercial real estate indicators and predictive analytics for the real estate market during the COVID-19 pandemic ^42^ predominantly utilize machine learning models, big data algorithms, and social data analytics. These methodologies rely on computational processing capabilities to analyze extensive datasets, monitor human behavior, and effectively predict market trends ( Figure 21).

Machine dependency in the context of forecasting commercial real estate indicators and predictive analytics during the COVID-19 pandemic ^43^ refers to the reliance on specific technological platforms, software applications, or algorithms for analyzing and interpreting large volumes of data. As the real estate market navigates the challenges posed by the pandemic, this dependency can significantly impact the accuracy and effectiveness of predictive models and insights.

**Figure 22.**
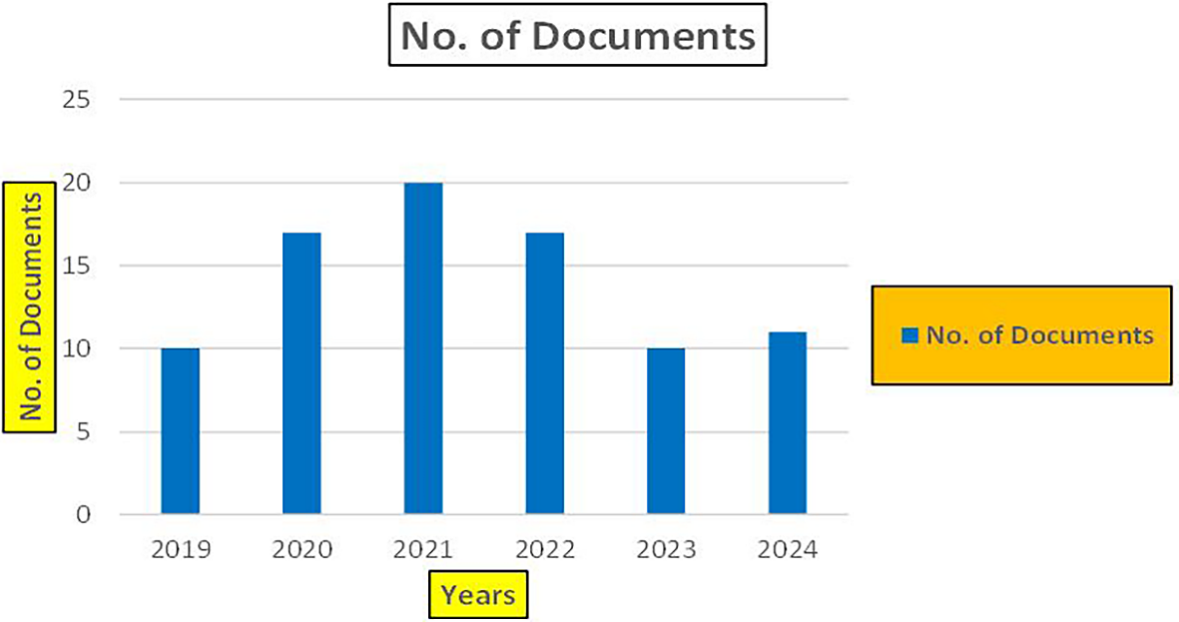
Real Estate Research Publications (2019-2024): Number of Papers Published.

For instance, when leveraging social big data to forecast commercial real estate indicators, researchers and analysts often depend on specific data processing frameworks or machine learning algorithms optimized for particular types of hardware. This dependency can create barriers to integrating diverse data sources, such as social media activity, economic indicators, and market trends. If the analytical models are designed around specific technological constraints, it can limit flexibility, making it difficult to adapt to changing market conditions or incorporate emerging data streams relevant to understanding human behavior during the pandemic ( Figure 22).

**Figure 23.**
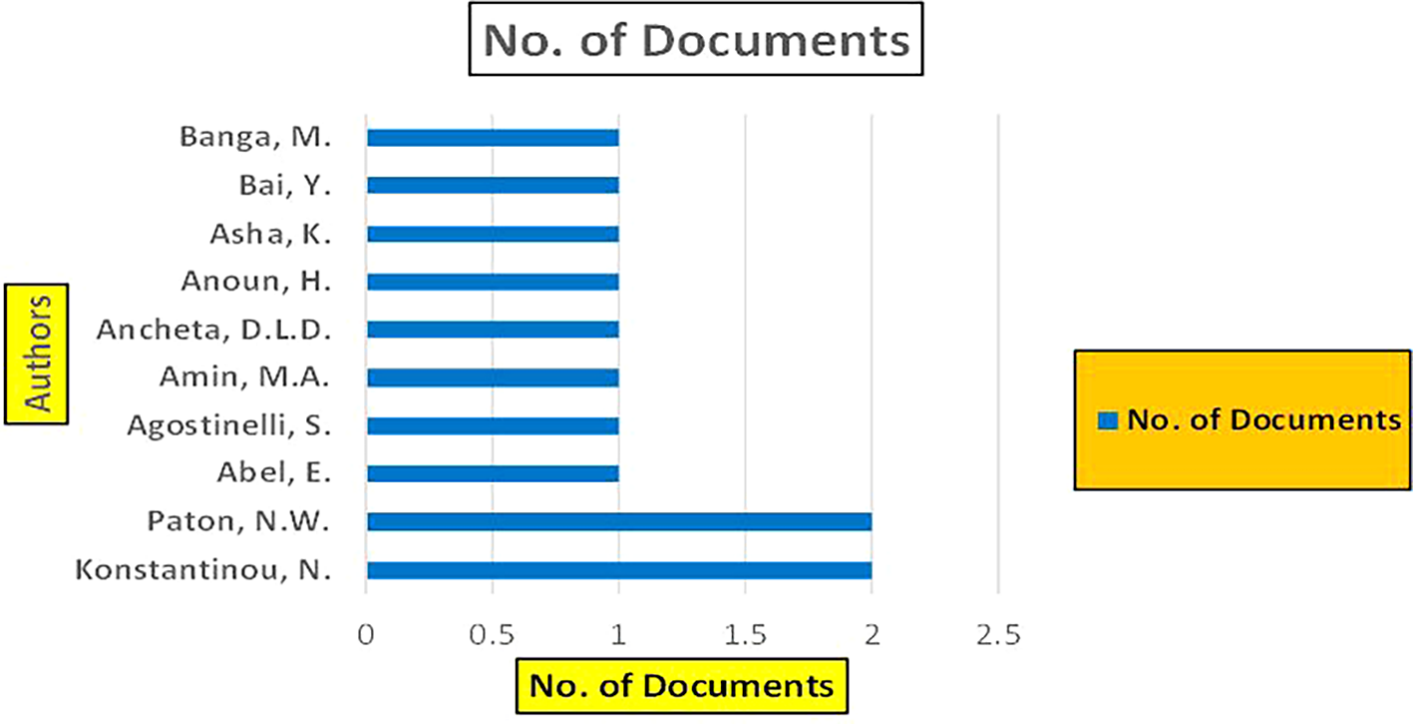
Real Estate Research Papers by Affiliation (2019-2024): Publication Trends.

Similarly, predictive analytics using big data for the real estate market may involve the use of proprietary software or tailored machine learning solutions. These systems can be complex and require specialized knowledge to operate effectively. This machine dependency might hinder real estate firms’ ability to rapidly adjust their analytics strategies in response to evolving market dynamics or consumer behaviors influenced by COVID-19. For example, if a predictive model is entrenched in a specific platform, switching to a more advanced or efficient system could entail significant costs and downtime, limiting the firm’s responsiveness to the fluidity of the real estate landscape ( Figure 23).

**Figure 24.**
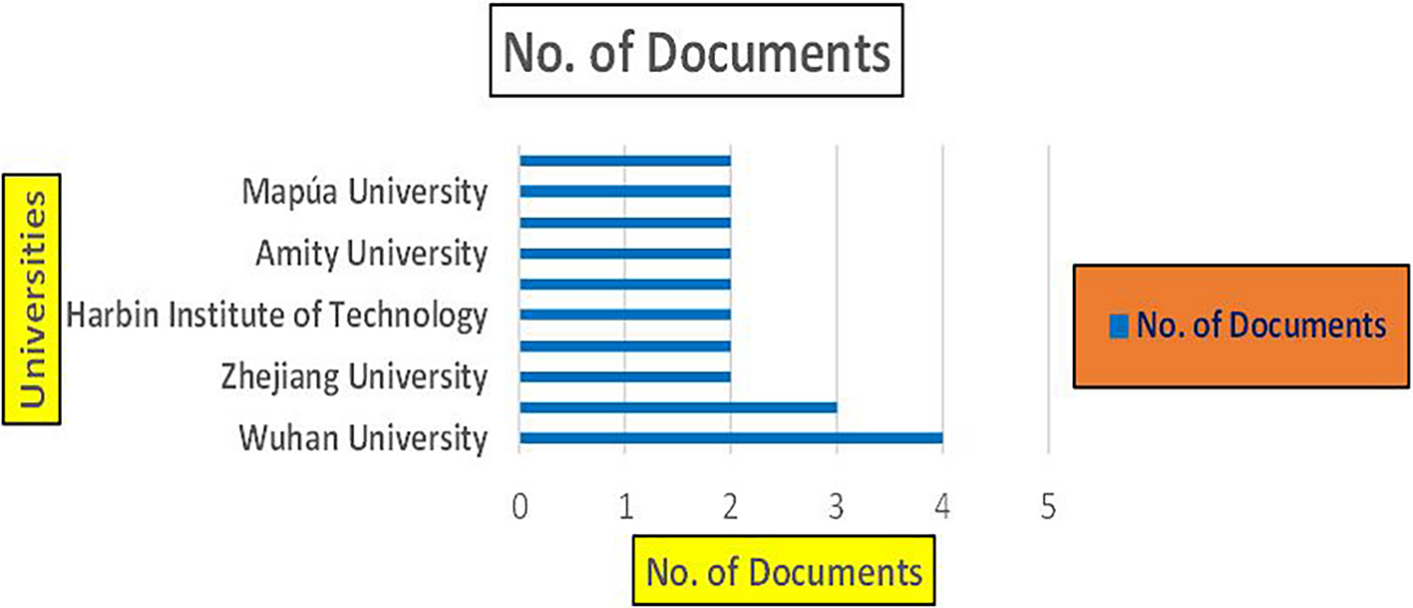
Real Estate Research Papers by Universities (2019-2024): Publication Trends.

Furthermore, the reliance on specific data processing and analytics tools can create a homogenized approach to data interpretation, leading to potential blind spots. When all analyses are conducted through a single technological lens, real estate analysts may miss critical insights that could arise from utilizing alternative methods or platforms. This is particularly relevant during a pandemic when market conditions are volatile, and the ability to pivot and adopt new methodologies quickly is paramount ( Figure 24).

Moreover, machine dependency can pose risks related to data privacy and security. Relying on specific systems may increase vulnerabilities, especially when handling sensitive data related to human activity and real estate transactions. If these systems are compromised, the implications can be severe, not only for individual firms but also for the broader market.

**Figure 25.**
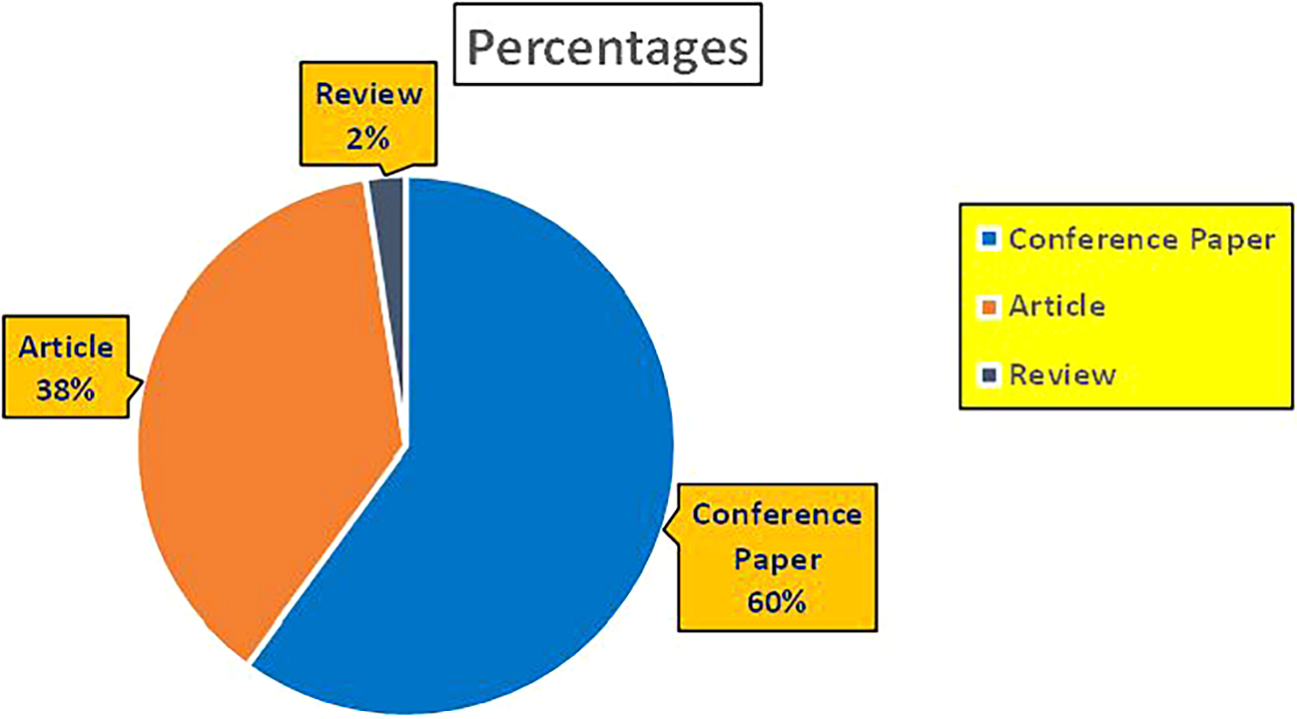
Real Estate Research Papers by Source (2019-2024): Publication Trends.

In conclusion, while big data analytics offers valuable tools for forecasting and predictive modeling in commercial real estate, machine dependency presents significant challenges. The reliance on specific technologies can inhibit flexibility, limit innovation, and create risks that may impede effective decision-making during uncertain times, such as the COVID-19 pandemic. To enhance adaptability and maintain a competitive edge, real estate firms should prioritize machine-independent solutions that enable more agile and comprehensive analysis ( Figure 25).

### 2.6 Machine dependency in Agricultural

Machine dependency on big data analytics and machine learning models to automate disaster risk management ^44^ and operational decision-making.

**Figure 26.**
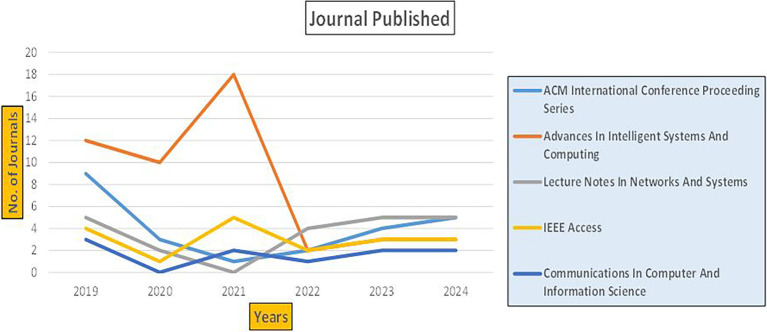
Agriculture Research Papers Published by Source (2019-2024).

Machine dependency in the context of agricultural disaster risk management and big data analytics applications in information management highlights the challenges associated with relying on specific technologies, software platforms, or algorithms to process and analyze large datasets. As these fields increasingly adopt big data analytics to enhance operational efficiencies and decision-making, the reliance on machines or systems can create barriers to flexibility, scalability, and innovation ( Figure 26).

**Figure 27.**
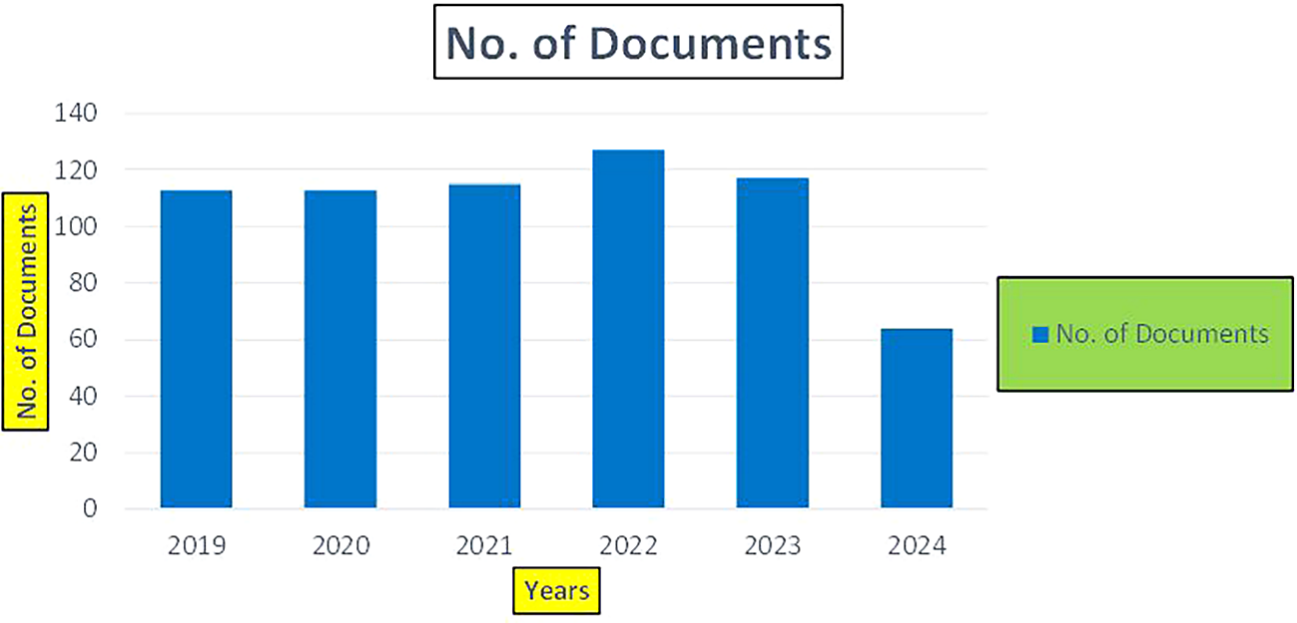
Agriculture Research Publications (2019-2024): Number of Papers Published.

In agricultural disaster risk management, big data analytics plays a crucial role in assessing risks, predicting events, and formulating response strategies. However, if these analytical processes are tied to specific hardware or proprietary software, the capacity to adapt to new data sources or methodologies can be significantly constrained. For instance, if a system is designed to analyze agricultural data exclusively through a particular cloud platform or machine learning framework, it may struggle to incorporate alternative data streams, such as real-time satellite imagery or sensor data from IoT devices. This can result in incomplete risk assessments and a diminished ability to respond effectively to emerging threats ( Figure 27).

**Figure 28.**
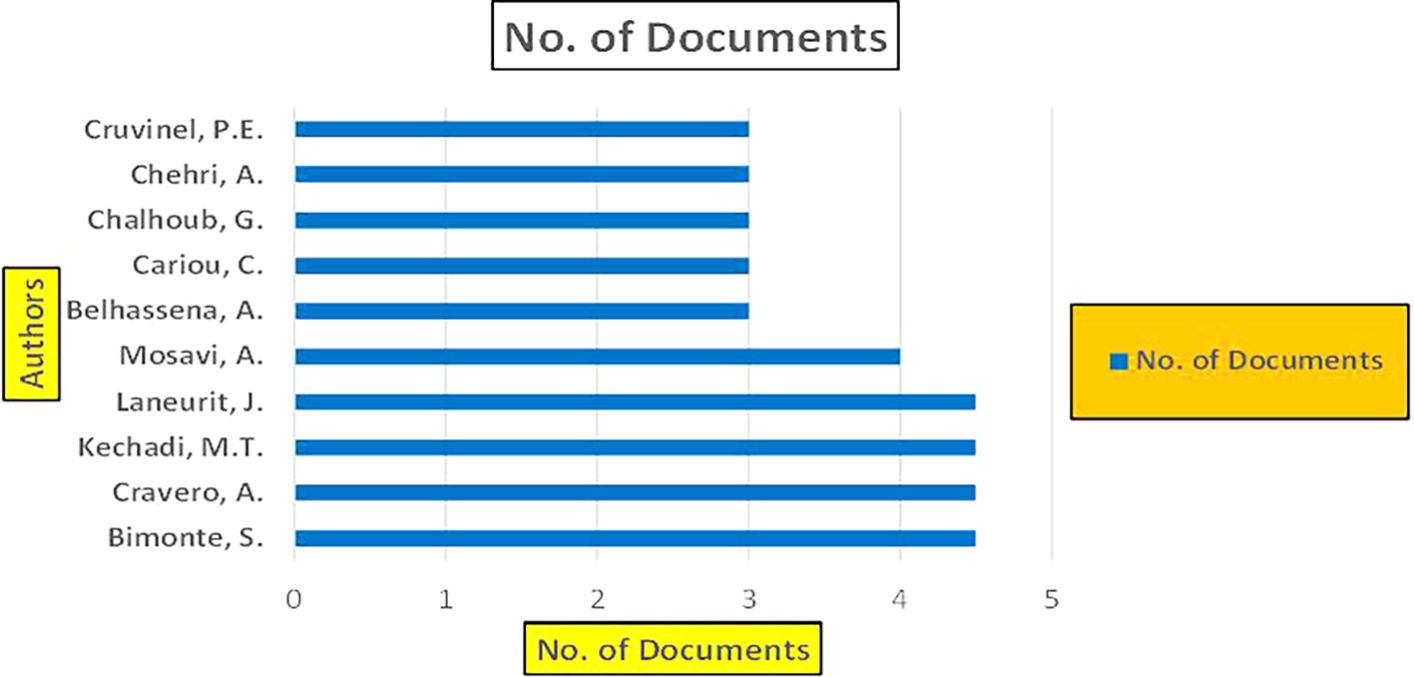
Agriculture Research Papers by Affiliation (2019-2024): Publication Trends.

Moreover, the bibliometric analysis of big data analytics applications in information management further illustrates machine dependency concerns. In this study, the reliance on specific statistical tools and programming languages, such as R, can limit the accessibility and reproducibility of the findings. Researchers may find themselves locked into particular analytical approaches, which can hinder the exploration of diverse methodologies or frameworks that could yield different insights. This dependency on certain technologies can stifle innovation and reduce the overall robustness of the research ( Figure 28).

**Figure 29.**
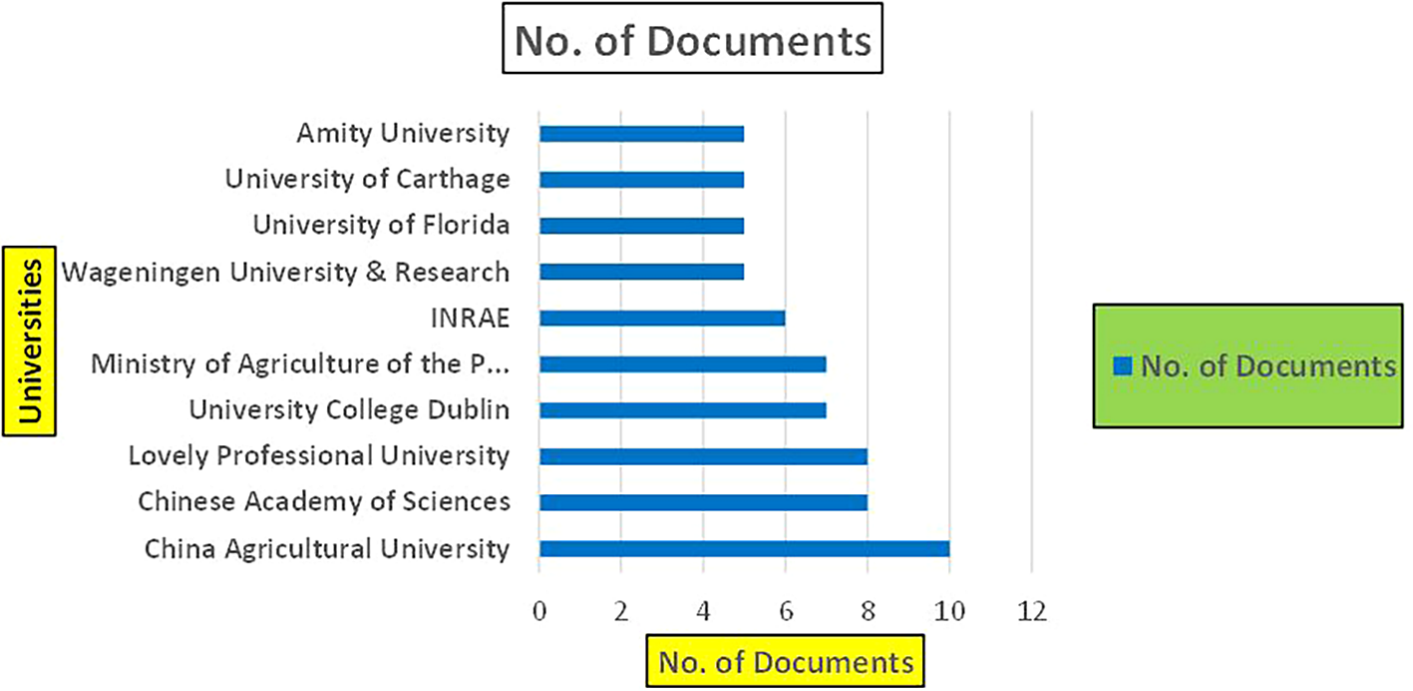
Agriculture Research Papers by Universities (2019-2024): Publication Trends.

Another critical aspect of machine dependency is the risk of vendor lock-in. If organizations commit to specific platforms for their big data analytics, they may face challenges when it comes time to upgrade or switch technologies. The associated costs and complexities of transitioning to new systems can lead to stagnation in their analytics capabilities. For example, agricultural organizations using specific big data tools may find it difficult to implement new analytical techniques or integrate cutting-edge technologies without incurring substantial costs or facing lengthy implementation bottlenecks ( Figure 29).

**Figure 30.**
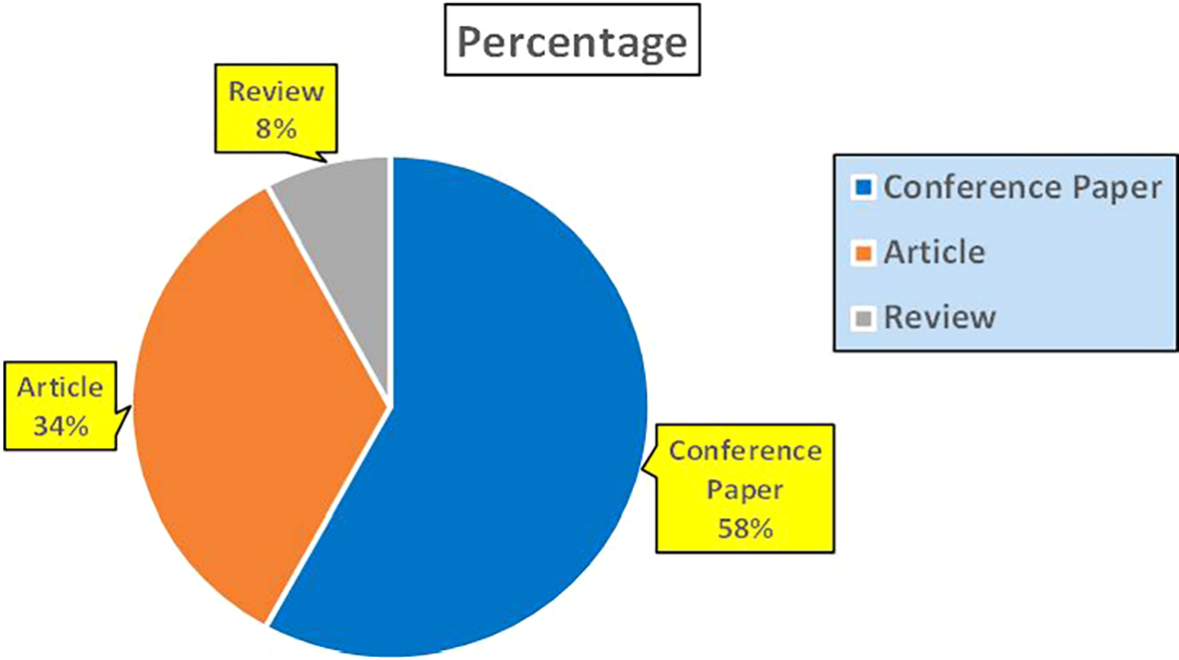
Agriculture Research Papers by Source (2019-2024): Publication Trends.

Furthermore, machine dependency can raise concerns about data security and privacy. When data is processed through specific machines or software, vulnerabilities may arise, particularly if those systems are not updated regularly or lack robust security measures. In agricultural settings, where data can include sensitive information about crop yields, market prices, ^45^ or resource allocations, the implications of such vulnerabilities can be **catastrophic**, potentially leading to data breaches or misuse ( Figure 30).

In summary, while big data analytics has transformative potential in agricultural disaster risk management and information management, machine dependency presents notable challenges. This reliance on specific technologies can limit flexibility, hinder innovation, and create risks that may undermine decision-making capabilities. To overcome these challenges, organizations should seek machine-independent solutions that promote interoperability and adaptability, ensuring they can effectively leverage big data analytics for improved outcomes in their respective fields.

## 3. Domain specific generation of huge amount of data

In this section the review research papers enumerated and explore diverse domains wherein substantial volumes of data are generated, particularly in healthcare, finance, retail, and agriculture. In the healthcare sector, ^46^ extensive data is generated through electronic health records (EHRs), ^47^ wearable sensors, medical imaging, and genomics. The finance industry contributes by processing large-scale transactional data, fraud detection patterns, and customer behavior analytics. In the retail sector, big data emerges from consumer purchasing patterns, inventory management, and social media influences. Agriculture generates substantial data through climate records, crop monitoring, and production analytics. These industries, collectively driven by real-time data acquisition and diverse sources, produce and manage colossal data volumes, rendering big data analytics essential for processing, prediction, and decision-making.

## 4. Data as a resource

### 4.1 As valuable insights in historical data

The afore mentioned review research papers elucidate the progressive role of big data analytics across various sectors, with particular emphasis on healthcare, finance, and retail. These fields have undergone a transition from conventional data management practices to the utilization of artificial intelligence, machine learning, and big data technologies for predictive analytics, risk management, and operational efficiency. The healthcare sector ^48^ focuses on disease prediction and electronic health record (EHR) management, ^49^ while the finance industry emphasizes fraud detection and credit risk assessment. The retail and agricultural sectors benefit from demand forecasting and disaster risk management, respectively, signifying substantial advancements in data-driven decision-making processes.

### 4.2 Data As a predictor for future data

This section presents a review of the data as a predictor for future based on current data across sectors:

**4.2.1 Healthcare:**

- **Disease prediction**: Advanced AI models will analyze genetic, environmental, and lifestyle data to predict individual disease risks with higher accuracy. ^50^
- **Supply chain efficiency**: Predictive analytics will optimize inventory management, reducing waste and ensuring timely delivery of medical supplies and pharmaceuticals.
- **Patientcare outcomes:** Machine learning algorithms will analyze treatment efficacy across diverse patient populations, enabling personalized medicine approaches.
- **Electronic health record trends:** Natural language processing will enhance the extraction of meaningful insights from unstructured medical data, improving clinical decision support systems
- **Risk assessment**: AI-driven tools will evaluate population health risks, enabling proactive public health interventions and resource allocation.

**4.2.2 Banking:**

- **Fraud detection:** Real-time anomaly detection systems will identify complex fraud patterns across multiple channels, reducing financial losses. ^51^
- **Credit risk assessment:** Advanced models will incorporate alternative data sources to provide more accurate and inclusive credit scoring.
- **Operational optimization:** AI-powered process ^52^ automation will streamline back-office operations, reducing costs and improving customer service.
- **Cybersecurity ^53^ threats:** Predictive models will anticipate emerging cyber threats, enabling proactive defense strategies.
- **Financial decision-making trends:** AI assistants will provide personalized financial advice based on individual spending patterns and market trends.

**4.2.3 Finance:**

- **Risk mitigation:** Sophisticated models will analyze global economic indicators to predict market volatility and optimize investment strategies.
- **Credit evaluation:** AI algorithms will assess creditworthiness using non-traditional data sources, expanding access to financial services.
- **Financial analysis:** Natural language processing will extract insights from financial reports and news, enhancing investment decision-making.
- **Sustainability assessment:** AI tools will evaluate companies’ environmental, social, and governance (ESG) performance, influencing investment choices. ^54^
- **Digital finance trends:** Predictive models will forecast the adoption and impact of emerging technologies like blockchain and decentralized finance. ^55^

**4.2.4 Retail:**

- **Sales trends:** AI-driven demand forecasting will optimize inventory management and pricing strategies across multiple channels.
- **Customer behavior:** Advanced analytics ^56^ will predict individual customer preferences, enabling hyper-personalized marketing and product recommendations.
- **Fraud detection:** Machine learning models will identify fraudulent transactions and returns in real-time, reducing losses. ^57^
- **Productivity optimization:** AI-powered workforce management systems will predict staffing needs and optimize employee scheduling.
- **External factor impacts:** Predictive models will assess the influence of economic, social, and environmental factors on consumer behavior and sales performance.

**4.2.5 Real Estate:**

- **Market trends during crises:** AI models will analyze historical data and current economic indicators to predict property value fluctuations during economic downturns or global events.
- **Location-based valuation:** Machine learning algorithms will incorporate diverse data sources (e.g., urban development plans, climate change projections) to predict long-term property value trends. ^58^
- **Rental market dynamics:** Predictive models will forecast rental demand and pricing trends, considering factors like remote work adoption and demographic shifts.
- **Investment risk assessment:** AI-driven tools will evaluate potential risks and returns for real estate investments across different property types and locations.
- **Sustainability impact:** Predictive analytics will assess the impact of energy efficiency and sustainability features on property values and market demand.

**4.2.6 Agriculture:**

- **Operational efficiency:** AI-powered systems will optimize resource allocation, including water usage, fertilizer application, and machinery deployment.
- **Disaster risk management:** Predictive models will forecast extreme weather events and pest outbreaks, enabling proactive mitigation strategies.
- **Crop yield projections:** Machine learning algorithms will analyze soil conditions, weather patterns, and genetic data to predict crop yields with higher accuracy. ^59^
- **Resource allocation:** AI-driven decision support systems will optimize the allocation of land, water, and labor resources based on market demand and environmental factors.
- **Precision agriculture:** Advanced analytics will enable highly targeted interventions at the individual plant level, maximizing yields while minimizing resource use.
- **Supply chain optimization:** Predictive models will forecast global agricultural supply and demand, informing planting decisions and reducing food waste.

This resource delineates domains wherein big data analytics, artificial intelligence, and machine learning can generate predictive insights across sectors.

## 5. Need of the hour survey for various applications of Data Analytics

The survey paper in question presents a comprehensive analysis of data analytics applications across multiple key sectors, drawing insights from over 1,000 research papers. By focusing on 100 of these papers, the investigation provides a systematic classification of research into various domains, specifically Healthcare, Banking, Finance, Retail, Real Estate, Agriculture, and Credit Card Fraud. Each of these sectors utilizes data analytics in distinct ways, and this survey elucidates the trends and innovations shaping the implementation of data-driven technologies in these fields.

## 6. Key domains

### 6.1 Healthcare

Data analytics is applied to enhance patient outcomes, optimize hospital operations, and advance personalized medicine. Big data in healthcare ^60^ is transforming the utilization of patient data, electronic health records[3], and medical research to improve diagnosis, treatment, ^62^ and prevention strategies.

### 6.2 Banking and finance

In banking and financial services, data analytics is extensively utilized ^63^ for risk management, fraud detection, customer profiling, and predictive modeling. Through the analysis of large datasets, financial institutions can forecast market trends, enhance decision-making processes, and improve compliance measures. ^64^

### 6.3 Retail

The retail sector employs data analytics to optimize supply chains, enhance customer experiences, and refine marketing strategies. Big data enables retailers to analyze purchasing patterns, predict demand, and personalize offers, thereby driving increased sales and operational efficiencies. The retail focuses on analyzing the spatial distribution and clustering of urban retail industry using POI big data, transportation, GDP, and population metrics.

### 6.4 Real estate

In real estate, data analytics facilitates the forecasting of market trends, evaluation of property values, and assessment of investment risks. By utilizing predictive models and analyzing factors such as location, demographic shifts, and economic indicators, stakeholders can make more informed decisions regarding property transactions.

### 6.5 Agriculture

Data analytics plays a crucial role in modern agriculture by improving crop management, resource allocation, and disaster risk assessments. Through the utilization of big data, farmers can enhance productivity, reduce costs, and mitigate the effects of climate change.

### 6.6 Credit/Debit Card Fraud

Credit card fraud detection is a critical application of data analytics, wherein machine learning models analyze transaction patterns to identify fraudulent activity. Real-time analysis enables financial institutions to detect and prevent fraud, thereby safeguarding customers’ financial assets. ^65^

## 7. Discussion

This section presents a discussion along with graphical representations of research work done in various sectors emphasizing further the advent of data analytics artificial intelligence and machine learning as the new era of research and its penetration into varied sectors.

### 7.1 Healthcare

### 7.2 Banking

The above Figure 6 represents number of Research Paper of Banking Published during different source the year ( **2019-2024**) and Figure 7 represents number of Research Paper of Banking Published during the year ( **2019-2024**).

The above Figure 8 represents the number of Research Paper of Banking Published during the year ( **2019-2024**) By Affiliation and Figure 9 represents the number of Research Paper of Banking Published during the year ( **2019-2024**) By College.

The above Figure 10 represents the number of Research Paper of Banking Published during the year ( **2019-2024**) By Different Source.

### 7.3 Finance

Figure 11 represents number of Research Paper of Finance Published in different source during the year ( **2018-2024**) and Figure 12 represents number of Research Paper of Finance Published during the year ( **2018-2024**).

Figure 13 represents the number of Research Paper of Finance Published during the year ( **2018-2024**) By Affiliation and Figure 14 represents the number of Research Papers of Finance Published during the year ( **2018-2024**) By College.

Figure 15 represents the number of Research Paper of Finance Published during the year ( **2018-2024**) By Different Source.

### 7.4 Retail

Figure 18 represents the number of Research Paper of Finance Published during the year ( **2019-2024**) By Affiliation and Figure 19 represents the number of Research Paper of Retail Published during the year ( **2019-2024**) By College.

The above Figure 20 represents the number of Research Paper of Retail Published during the year ( **2019-2024**) By Different Source.

### 7.5 Real Estate

Figure 21 represents number of Research Paper of Real Estate Published during different source of the year ( **2019-2024**) and Figure 22 represents number of Research Paper of Real Estate Published during the year ( **2019-2024**).

Figure 23 represents the number of Research Paper of Real Estate Published during the year ( **2019-2024**) By Affiliation and Figure 24 represents the number of Research Paper of Real Estate Published during the year ( **2019-2024**) By College.

Figure 25 represents the number of Research Paper of Real Estate Published during the year ( **2019-2024**) By Different Source.

The above Figure 26 represents number of Research Paper of Agriculture Published in different source during the year ( **2019-2024**) and Figure 27 represents number of Research Paper of Agriculture Published during the year ( **2019-2024**).

## Conclusion

This survey highlights the diverse and impactful ways data analytics is being applied across various sectors. Each domain benefits from tailored approaches to handling big data, driving innovation, and improving operational efficiency. The classification of research papers into these domains offers a structured overview, guiding further exploration into how data analytics continues to evolve and address critical challenges across industries.

## Ethics and consent

No ethics and consent were required.

## Data Availability

No data are associated with this article. Zenodo repository: PRISMA checklist for ‘Synergistic review of automation impact of big data, AI, and ML in current data transformative era. https://doi.org/10.6084/m9.figshare.28375625.v1 ^66^ Data are available under the terms of the Creative Commons Zero “No rights reserved” data waiver (CC0 1.0 Public domain dedication).
