# Supplementary material for: Synergistic review of automation impact of big data, AI, and ML in current data transformative era
Source: F1000Res. 2025 May 22;14:253. Originally published 2025 Mar 3. [Version 2] doi: 10.12688/f1000research.161477.2 (PMC12296276; doi:10.12688/f1000research.161477.2)
Supplement: Peer review history

# Reviewer report: Reviewer response for version 2

**DOI:** 10.5256/f1000research.181687.r390987

**Published:** 2025-07-26

**Authors:** Edwin Ramirez Asis

**Affiliations:** 1 Pontifical Catholic University of Peru, San Miguel, Peru

The article addresses the topic from a scientific research perspective, and version 2 has addressed some shortcomings. It is accepted for indexing.

 Regarding the review, the classification of research articles into these domains provides a structured overview that guides exploration of how data analysis continues to evolve and address critical challenges in different industries. Furthermore, the use of Jamovi, Exploratory Factor Analysis (EFA), and regression analysis illustrate the application of robust quantitative methods, providing a structured, data-driven approach to exploring complex research topics.

Are the rationale for, and objectives of, the Systematic Review clearly stated?

Yes

Is the statistical analysis and its interpretation appropriate?

Yes

If this is a Living Systematic Review, is the ‘living’ method appropriate and is the search schedule clearly defined and justified? (‘Living Systematic Review’ or a variation of this term should be included in the title.)

Yes

Are sufficient details of the methods and analysis provided to allow replication by others?

Yes

Are the conclusions drawn adequately supported by the results presented in the review?

Yes

Reviewer Expertise:

I hold a PhD in business administration, am a university professor with over 20 years of experience, and have 49 articles published in Scopus.

I confirm that I have read this submission and believe that I have an appropriate level of expertise to confirm that it is of an acceptable scientific standard.

# Reviewer report: Reviewer response for version 2

**DOI:** 10.5256/f1000research.181687.r386651

**Published:** 2025-06-02

**Authors:** Geeta Sandeep Nadella

**Affiliations:** 1 University of the Cumberlands, Williamsburg, Kentucky, USA

Areas for Improvement  

  1. Clarity of Objectives and Rationale  

 - Issue: The rationale lacks explicit justification for the review’s synergistic focus. Why is a cross-sector analysis necessary? How does this fill gaps left by sector-specific reviews?  

 - Fix:  

   - Clearly state the review’s unique contribution (e.g., identifying common challenges like machine dependency across sectors).  

   - Define specific research questions (e.g., "How do AI/ML adoption barriers differ between healthcare and finance?").  

  2. Statistical and Analytical Rigor  - Only descriptive stats are present, but no inferential stats. This is a crtical issue.

 - Issue: Methods like EFA and regression are mentioned but lack context. Are these applied to the reviewed studies or original data? Assumptions (e.g., normality) and effect sizes (e.g., β-values) are unaddressed.  

 - Fix:  

   - Clarify the purpose of statistical tools (e.g., "EFA was used to categorize challenges").  

   - Include meta-analysis (if feasible) to quantify trends (e.g., "AI improved fraud detection accuracy by 30% in 15/20 studies").  

  4. Critical Analysis and Synthesis  - Section 6, 7 and 8 are poorly written. add the actual analysis and discussion in there, not just pointing out to the pictures.

 - Issue: The discussion is descriptive rather than analytical. For example:  

   - Machine dependency in healthcare is discussed but not compared to challenges in retail (e.g., vendor lock-in vs. scalability).  

   - Ethical considerations (e.g., data privacy) are mentioned but not linked to sector-specific case studies.  

 - Fix:  

   - Add a comparative table summarizing cross-sector challenges (e.g., "Healthcare: Interoperability vs. Retail: Vendor lock-in").  

   - Discuss limitations of existing studies (e.g., bias toward high-income countries in agricultural data).  

  5. Presentation and Formatting  

 - Issue:  

   - Mislabeled figures (e.g., Table 6 labeled as "Digital security culture" instead of XDR).  

   - Inconsistent citation formatting (e.g., missing DOIs in some references).  

 - Fix:  

   - Standardize table/figure labels (e.g., "Figure 6: Extended Detection and Response (XDR)").  

   - Ensure all references follow journal guidelines (e.g., italicize journal names, include URLs/DOIs).  

  6. Conclusion and Implications  

 - Issue: Conclusions restate findings without actionable recommendations (e.g., "machine-independent solutions are needed" lacks sector-specific guidance).  

 - Fix:  

   - Propose solutions (e.g., "Adopt open-source frameworks in healthcare to reduce vendor lock-in").  

   - Highlight future research directions (e.g., "Explore AI ethics in agricultural disaster management").  

 ---

  Recommendations for Revision  

 1. Restructure the Introduction: Explicitly state gaps in existing literature and the review’s unique value.  

 2. Expand Methods Section: Detail search strategies, inclusion/exclusion criteria, and data synthesis protocols.  

 3. Enhance Critical Analysis: Compare sector-specific challenges and synthesize trends (e.g., "Data quality issues persist across all sectors").  

 4. Correct Presentation Errors: Ensure accurate labeling of figures/tables and consistent citation formatting.  

 5. Strengthen Conclusions: Link findings to practical implications (e.g., "Policymakers should prioritize interoperable frameworks").  

 ---

  Final Remarks  

 This review has significant potential to inform interdisciplinary research and practice. Addressing methodological transparency, deepening critical analysis, and refining presentation will elevate its impact. Consider collaborating with a methodologist or statistician to strengthen quantitative synthesis.

Are the rationale for, and objectives of, the Systematic Review clearly stated?

Partly

Is the statistical analysis and its interpretation appropriate?

Partly

If this is a Living Systematic Review, is the ‘living’ method appropriate and is the search schedule clearly defined and justified? (‘Living Systematic Review’ or a variation of this term should be included in the title.)

Not applicable

Are sufficient details of the methods and analysis provided to allow replication by others?

Partly

Are the conclusions drawn adequately supported by the results presented in the review?

Partly

Reviewer Expertise:

AI, ML, Systems Analysis, Theoretical designs, Big Data, Cybersecurity, Information Systems Management.

I confirm that I have read this submission and believe that I have an appropriate level of expertise to confirm that it is of an acceptable scientific standard, however I have significant reservations, as outlined above.

# Reviewer report: Reviewer response for version 1

**DOI:** 10.5256/f1000research.177513.r371087

**Published:** 2025-04-28

**Authors:** Narayanage Jayantha Dewasiri

**Affiliations:** 1 Sabaragamuwa University of Sri Lanka, Belihuloya, Sri Lanka

Thank you for your contribution. Your manuscript has a serious flaw in the methodology. Please address the followings. 

 Please apply PRISMA and PICO approaches under a separate section called Methodology which outlines the scientific approach that you have taken for this study. You can cite the below papers to support the use of PRISMA and PICO approaches as the methodology.

 Please see [Ref 1 to 6].

Are the rationale for, and objectives of, the Systematic Review clearly stated?

Yes

Is the statistical analysis and its interpretation appropriate?

Yes

If this is a Living Systematic Review, is the ‘living’ method appropriate and is the search schedule clearly defined and justified? (‘Living Systematic Review’ or a variation of this term should be included in the title.)

Yes

Are sufficient details of the methods and analysis provided to allow replication by others?

Partly

Are the conclusions drawn adequately supported by the results presented in the review?

Yes

Reviewer Expertise:

Management & AI

I confirm that I have read this submission and believe that I have an appropriate level of expertise to confirm that it is of an acceptable scientific standard.

## References

[1] : Structured Exercise Program for Hip Arthroplasty: An Expert Consensus Using the Delphi Technique. *Indian J Orthop* .2025;59(4) : 10.1007/s43465-025-01335-3 539-548 10.1007/s43465-025-01335-3 40276788 PMC12014874

[2] : Structured Exercise Program for Hip Arthroplasty: An Expert Consensus Using the Delphi Technique. *Indian J Orthop* .2025;59(4) : 10.1007/s43465-025-01335-3 539-548 10.1007/s43465-025-01335-3 40276788 PMC12014874

[3] : Transforming Healthcare Services in South Asia.2025; 10.1201/9781003483113-4 63-75 10.1201/9781003483113-4

[4] : Challenges of Integrating Social Responsibility and Climate Change for the Sustainable Development Goals: Experience From the South Asian Context. 10.1108/S2043-052320250000025011 205-226 10.1108/S2043-052320250000025011

[5] : Highlights of Circular Economy Actions in the Climate Change Policies: A Call for Action for Asia-Pacific Region. 10.1108/S2043-052320250000025002 23-41 10.1108/S2043-052320250000025002

[6] : Structured Exercise Program for Hip Arthroplasty: An Expert Consensus Using the Delphi Technique. *Indian J Orthop* .2025;59(4) : 10.1007/s43465-025-01335-3 539-548 10.1007/s43465-025-01335-3 40276788 PMC12014874

# Response

**Published:** 2025-05-06

**Authors:** Swastik Rath

**Affiliations:** Computer Science & Engg., Kalinga Institute of Industrial Technology, Bhubaneswar, Odisha, India

Respected Sir,

 #1 - Prisma Flow diagram is uploaded on the Methodological Section of the Systematic Review Paper in new version 2.

 #2 - All the papers that are mentioned are cited in the new version 2.

 Yours faithfully

  Swastik Rath

# Reviewer report: Reviewer response for version 1

**DOI:** 10.5256/f1000research.177513.r377823

**Published:** 2025-04-26

**Authors:** Geeta Sandeep Nadella

**Affiliations:** 1 University of the Cumberlands, Williamsburg, Kentucky, USA

#1 The systematic review partially clarifies its rationale and objectives but requires refinement for greater precision. The rationale broadly highlights the transformative role of automation, big data, AI, and ML across six sectors (e.g., healthcare, finance) and acknowledges challenges like data quality and privacy. However, it lacks explicit justification for its synergistic focus, failing to address gaps in existing literature (e.g., fragmented sector-specific reviews) or the urgency of cross-sector analysis. The objectives outline the intent to explore technological impacts and list applications (e.g., fraud detection in banking) but are overly vague, omitting structured goals (e.g., comparative analyses of adoption barriers) and methodological rigor (e.g., undefined study selection criteria).

 To enhance clarity, the review should explicitly state its unique contribution (e.g., synthesizing cross-sector insights), structure objectives as distinct research questions, and define scope (e.g., peer-reviewed studies from 2018–2025). Methodological transparency, such as a PRISMA flowchart and justification for sector inclusion, would strengthen its systematic approach. While the review’s intent is evident, sharper articulation of gaps, objectives, and methods would bolster its academic rigor and practical relevance.

 #2 The systematic review partly provides sufficient methodological details, with notable strengths and weaknesses. Strengths include a broad foundation of 1,000+ reviewed papers, focused analysis of 100 key studies, and visual aids (e.g., figures on publication trends) to illustrate sector-specific research activity. Analytical tools like Jamovi and statistical methods (EFA, regression) are referenced, suggesting technical rigor. However, weaknesses undermine reproducibility: the absence of a PRISMA flow diagram obscures study selection processes; inclusion/exclusion criteria for selecting the 100 studies are undefined; search strategies (databases, keywords, timeframe justification) are omitted; and data synthesis methods (e.g., theme identification like "machine dependency") lack clarity. Additionally, statistical protocols (e.g., variable selection, regression assumptions) are inadequately described.

 To improve, the review should adopt standard systematic review practices:

 Include a PRISMA flowchart to detail screening stages (identification, screening, eligibility, inclusion).

 Define search strings (e.g., "big data AND healthcare AND machine learning") and inclusion/exclusion criteria (e.g., peer-reviewed articles from 2018–2025).

 Clarify data extraction (e.g., coding frameworks for sector/thematic categorization) and statistical rationale (e.g., why EFA was chosen over other methods).

 In conclusion, while the review’s scope and analytical tools are commendable, critical methodological gaps hinder replication. Enhancing transparency in search strategies, study selection, and analysis protocols would strengthen rigor and ensure reproducibility. Addressing these issues would align the review with systematic standards and bolster its academic credibility.

 #3 The statistical analysis in the systematic review is partially appropriate. Appropriate aspects include the use of validated methods like Exploratory Factor Analysis (EFA) with factor loadings >0.7 and Cronbach’s α >0.7 to assess reliability, as well as regression analysis to quantify relationships (e.g., financial constraints β = 0.604). Descriptive statistics, such as demographic data (e.g., 67% low literacy) and publication trends, are also clearly presented. These methods align with standard practices for analyzing survey or primary data.

 However, key weaknesses limit the analysis. The application of EFA and regression is unconventional for systematic reviews unless synthesizing primary data, and the manuscript does not clarify whether these methods were applied to the reviewed studies or a separate dataset. Critical assumption checks (e.g., normality, multicollinearity) are missing, and results lack contextual interpretation (e.g., effect sizes or practical implications of β values). Additionally, the absence of meta-analysis—a hallmark of systematic reviews—undermines quantitative synthesis of findings across studies.

 To improve, the review should clarify the methodology (e.g., specify if statistical tests were applied to reviewed studies or original data), validate assumptions (e.g., residual analysis for regression), and contextualize results (e.g., discuss effect sizes like Cohen’s f²). Adopting PRISMA guidelines, conducting meta-analyses, and justifying analytical choices would enhance rigor. While the statistical techniques are technically sound, their unconventional application and lack of transparency limit reproducibility and alignment with systematic review standards. Strengthening these areas would ensure the analysis meets academic expectations.

 #4 The systematic review’s conclusions are partly supported by the presented results. Supported conclusions include sector-specific applications of AI/ML (e.g., fraud detection in banking, disease prediction in healthcare), validated by figures showing publication trends and case studies. The discussion of machine dependency in sectors like healthcare and agriculture is also substantiated, with examples highlighting scalability and interoperability challenges. However, several conclusions lack robust evidence. Claims about the transformative impact of AI/ML are overstated, as the review does not quantify outcomes (e.g., healthcare efficacy improvements) or critically assess real-world adoption. Challenges like data quality and ethics are mentioned but not deeply explored, failing to link ethical concerns to sector-specific examples. Recommendations for machine-independent solutions or future directions lack empirical backing from the reviewed studies.

 Key gaps weaken the review’s credibility. There is no comparative analysis of AI/ML adoption barriers across sectors (e.g., healthcare vs. retail), undermining claims of cross-sector synergies. The review superficially addresses limitations like data privacy, without synthesizing evidence on their prevalence or severity. Overreliance on publication metrics (e.g., paper counts) conflates research activity with technological impact, ignoring study quality or practical implementation. For instance, trends in healthcare publications (Figure 1) do not confirm whether AI tools are clinically effective or widely adopted.

 To strengthen conclusions, the review should integrate sector-specific evidence (e.g., “AI improved diagnostic accuracy in 15/20 healthcare studies”) and employ meta-analytic techniques to quantify trends (e.g., average fraud detection accuracy gains). Challenges and future directions must be explicitly tied to findings (e.g., agricultural machine dependency justifying interoperable solutions). Addressing these gaps through deeper synthesis and critical appraisal would enhance the review’s rigor and credibility, ensuring conclusions are both data-driven and actionable.

Are the rationale for, and objectives of, the Systematic Review clearly stated?

Partly

Is the statistical analysis and its interpretation appropriate?

Partly

If this is a Living Systematic Review, is the ‘living’ method appropriate and is the search schedule clearly defined and justified? (‘Living Systematic Review’ or a variation of this term should be included in the title.)

Not applicable

Are sufficient details of the methods and analysis provided to allow replication by others?

Partly

Are the conclusions drawn adequately supported by the results presented in the review?

Partly

Reviewer Expertise:

AI, ML, Systems Analysis, Theoretical designs, Big Data, Cybersecurity, Information Systems Management.

I confirm that I have read this submission and believe that I have an appropriate level of expertise to confirm that it is of an acceptable scientific standard, however I have significant reservations, as outlined above.

# Response

**Published:** 2025-05-06

**Authors:** Swastik Rath

**Affiliations:** Computer Science & Engg., Kalinga Institute of Industrial Technology, Bhubaneswar, Odisha, India

Respected Sir,

 The changes that you suggested to do in the manuscript in done in the new version

 #1- This change was done and attached after the Last part of the Introduction.

 #2- The Prisma Flow flow chart is uploaded on the Methodological Section of the Systematic Review Paper in version 2

 #3 - Country-wise documents percentage graph published  in the new version from Figure No. 31 to Figure No. 36

 #4 - The modification of the conclusion part is modified and attached at the last of the conclusion

 There are a few modifications in the graphs in the new version of the manuscript.

 #3- The country-wise document percentage graphs of all the different sectors are shown in the last figures of all the sectors.

 Yours Faithfully

 Swastik Rath
